# TCA and SSRI Antidepressants Exert Selection Pressure for Efflux-Dependent Antibiotic Resistance Mechanisms in Escherichia coli

**DOI:** 10.1128/mbio.02191-22

**Published:** 2022-11-14

**Authors:** Jin Ou, Paul Elizalde, Hao-Bo Guo, Hong Qin, Brian T.D. Tobe, John S. Choy

**Affiliations:** a Department of Biology, The Catholic University of America, Washington, DC, USA; b Department of Computer Science and Engineering, University of Tennessee at Chattanooga, Chattanooga, Tennessee, USA; c Department of Psychiatry, Southern California Kaiser Permanente Medical Group, San Diego, California, USA; Louis Stokes Veterans Affairs Medical Center

**Keywords:** *E. coli*, SSRI, TCA, antibiotics, antidepressants, drug resistance mechanisms, efflux pumps, gut microbiota

## Abstract

Microbial diversity is reduced in the gut microbiota of animals and humans treated with selective serotonin reuptake inhibitors (SSRIs) and tricyclic antidepressants (TCAs). The mechanisms driving the changes in microbial composition, while largely unknown, is critical to understand considering that the gut microbiota plays important roles in drug metabolism and brain function. Using Escherichia coli, we show that the SSRI fluoxetine and the TCA amitriptyline exert strong selection pressure for enhanced efflux activity of the AcrAB-TolC pump, a member of the resistance-nodulation-cell division (RND) superfamily of transporters. Sequencing spontaneous fluoxetine- and amitriptyline-resistant mutants revealed mutations in marR and lon, negative regulators of AcrAB-TolC expression. In line with the broad specificity of AcrAB-TolC pumps these mutants conferred resistance to several classes of antibiotics. We show that the converse also occurs, as spontaneous chloramphenicol-resistant mutants displayed cross-resistance to SSRIs and TCAs. Chemical-genomic screens identified deletions in marR and lon, confirming the results observed for the spontaneous resistant mutants. In addition, deletions in 35 genes with no known role in drug resistance were identified that conferred cross-resistance to antibiotics and several displayed enhanced efflux activities. These results indicate that combinations of specific antidepressants and antibiotics may have important effects when both are used simultaneously or successively as they can impose selection for common mechanisms of resistance. Our work suggests that selection for enhanced efflux activities is an important factor to consider in understanding the microbial diversity changes associated with antidepressant treatments.

## INTRODUCTION

Selective serotonin reuptake inhibitors (SSRIs) and tricyclic antidepressants (TCAs) are used to treat a variety of psychiatric disorders and are among the most highly prescribed drugs in the United States (1). SSRIs and TCAs are best known for their action in regulating neuronal reuptake of synaptic monoamines (2, 3). Although it is well established that SSRIs and TCAs inhibit monoamine reuptake transporters there remains large gaps in a full understanding of how these drugs mitigate depression and other psychiatric disorders (4, 5). Furthermore, it is unclear what factors contribute to patient responses such as treatment-resistance or side effects leading to discontinued use (6–9). In addition to acting as inhibitors of monoamine reuptake transporters, SSRIs and TCAs are known to have antimicrobial, antifungal, and antiviral activities (10–15). Numerous studies have reported that animals and humans treated with SSRIs and TCAs experience changes in the composition of their gut microbiota (GM) raising the possibility that gut microbes may be important unintended targets of SSRIs and TCAs (12, 16–27). In turn, altered GM that occurs due to antibiotic treatment has been shown to be associated with a variety of behavioral and psychological disorders (28–33). Considering that the GM is known to play important roles in drug metabolism (34–36) and brain function (18, 37–39), these observations suggest that GM changes conferred by SSRI and TCA treatments may play important role(s) in treatment efficacy.

The TCA, amitriptyline (Elavil), and the SSRI, fluoxetine (Prozac) are two of the top 10 most frequently prescribed antidepressants (1, 40, 41) and both are known to have antimicrobial activity toward a variety of bacteria, including gut commensals (42–45). Yet, the mechanism(s) of SSRI and TCA antimicrobial activities and the extent of overlap with antibiotic resistance mechanism(s) remains largely unknown. Addressing these questions will be important to understand how these drugs interact with the GM and provide insights into how the GM effects may contribute to treatment outcomes. Studies of humans and animals treated with SSRIs and TCAs suggest that there may be some mechanisms of resistance in common with antibiotics as antibiotic resistance genes (ARGs) are found to be enriched in the GM (21, 25). Furthermore, Escherichia coli grown under chronic exposure to fluoxetine has been shown to increase mutagenesis and lead to mutants that develop antibiotic resistance (46). While these studies use different approaches and model systems the trend of observing an enrichment for ARGs suggest that SSRIs and TCAs may directly exert selection pressure that can overlap with antibiotics. Identifying these resistance mechanisms can advance our understanding of what drives the changes in GM diversity and what consequences they might have on treatment outcomes.

We used two approaches to determine if SSRIs and TCAs exert selection pressure for ARGs and the extent to which there may be overlap with antibiotic resistance mechanisms. In the first, we isolated spontaneous resistant E. coli mutants that were able to grow in the presence of lethal doses of fluoxetine or amitriptyline followed by whole-genome sequencing. Our second approach relied on chemical-genomic screens to identify resistant deletion mutants. We observed that a majority of spontaneous fluoxetine- and amitriptyline-resistant mutants simultaneously displayed resistance to antibiotics which target cell wall synthesis, translation, transcription, and replication. We found that all antidepressant-resistant mutants that displayed resistance to antibiotics also carried mutations in the transcriptional repressor MarR or the Lon protease. Both are important regulators of the AcrAB-TolC pump (47–49). Our chemical-genomic screens provided additional support that fluoxetine and amitriptyline select for mutants that increase AcrAB-TolC efflux activity as deletions in marR and lon were identified. Furthermore, the screens revealed 35 genes with diverse functions that provided resistance to SSRIs and TCAs and almost all conferred some degree of resistance to at least one antibiotic tested. Although this set of genes have no reported role in regulating efflux activity, we found many to be associated with increased efflux as measured by Hoechst accumulation assays. We also observed that spontaneous chloramphenicol resistance was associated with SSRI and TCA resistance. Together, these results support the idea that SSRI and TCA treatment can directly lead to enrichment for mutants with higher efflux activity and conversely, antibiotic exposure (e.g., chloramphenicol) may lead to changes that increase resistance to antidepressants. Our work suggests that selection for antibiotic resistance activities (e.g., AcrAB-TolC efflux) is an important factor to consider when addressing the question of how antidepressant treatments drive changes in microbial diversity. Furthermore, when both antidepressants and antibiotics are used simultaneously or successively, there may be important consequences on treatment outcomes as they can impose selection for common mechanisms of resistance.

## RESULTS

### Fluoxetine- and amitriptyline-resistant mutant E. coli display cross-resistance to related antidepressants.

**FIG 1:**
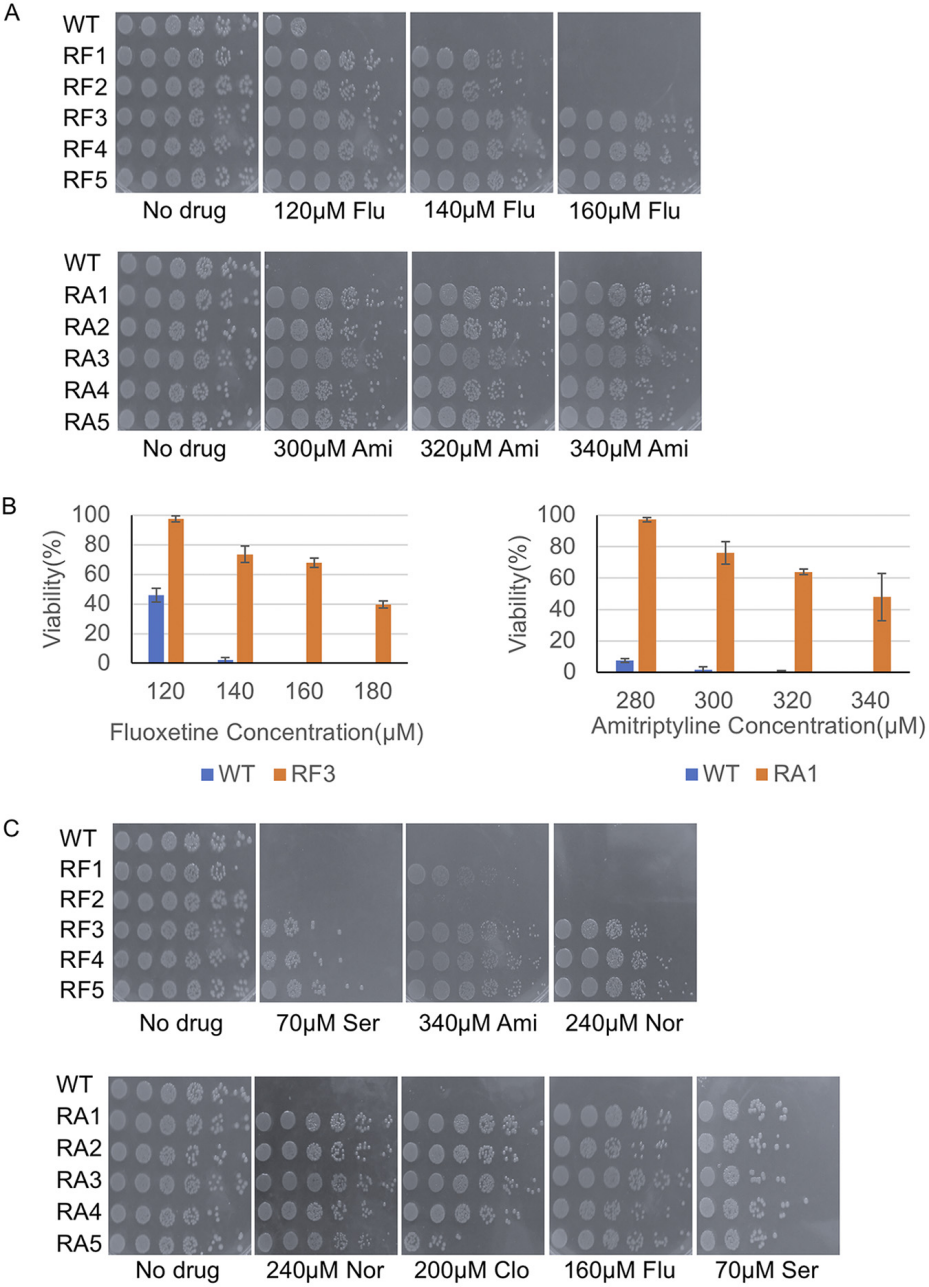
Isolation of spontaneously resistant mutants to fluoxetine and amitriptyline. Single colonies of E. coli were used to inoculate liquid cultures and allowed to grow overnight at 37°C. Cells were diluted and spread on plates containing fluoxetine or amitriptyline at MIC90. Colonies that grew on plates were isolated and served as candidate-resistant mutants which were subsequently tested in spot assays. (A) Spot tests of a subset of spontaneous mutants resistant to lethal doses of fluoxetine (RF) and amitriptyline (RA). RF1-5 and RA1-5 all display resistance to drug concentrations that are beyond the lethal dose for the parental strain (wild-type). (B) The viability of the wild-type, RF3, and RA1 strains was determined on increasing doses of fluoxetine (Flu) and amitriptyline (Ami). Single colonies of each strain were grown in overnight cultures, diluted, and spread on plates containing fluoxetine (120 to 180 μM), amitriptyline (280 to 340 μM), or no drug. Plates were incubated overnight at 37°C and colonies were counted. Plotted is the average percentage of viable cells (Viability %) calculated by dividing the number of CFU on drug plates by the CFU found on no drug control plates; error bars represent SEM for three independent cultures for each strain. (C) Spot tests of RF and RA mutants reveal cross-resistance to SSRIs (sertraline [Ser]) and TCAs (nortriptyline [Nor] and clomipramine [Clo]). For all spot tests single colonies of each strain were grown in overnight liquid cultures, diluted to an OD_600_ of 0.1, and 3 μL of serial 5-fold dilutions were spotted on the respective plates, incubated overnight at 37°C, and imaged.

To determine whether antidepressants exert selection pressure for mutations in genes that regulate efflux activity, we isolated spontaneously resistant mutants to fluoxetine and amitriptyline. We grew overnight cultures of E. coli in the absence of drug starting with single colonies and isolated resistant colonies that appeared on plates at ~MIC90 for fluoxetine and amitriptyline (Fig. S1B; Table S1). At least five colonies from each selection experiment were retested for resistance (Table S1). Each candidate-resistant colony was used to inoculate overnight cultures and 5-fold serial dilutions were spotted onto a set of plates with increasing doses of the drug used in the initial selection. All candidates from each drug selection experiment were more resistant to the drug than the parental strain but displayed a range of resistance (Fig. 1A). In almost all cases, candidates displayed resistance beyond the lethal dose used in the initial selection (Fig. 1A). At the highest dose of fluoxetine tested (180 μM) we observed no survivors from the parental strain but ~45% of RF3 mutants remained viable (Fig. 1B). Similarly, no survivors were observed from the parental strain at high doses of amitriptyline (340μΜ); nonetheless, ~60% of RA1 mutants remained viable (Fig. 1B).

10.1128/mbio.02191-22.1 TABLE S1 List of spontaneously resistant mutants to fluoxetine and amitriptyline. Download Table S1, DOCX file, 0.02 MB. Copyright © 2022 Ou et al. 2022 Ou et al. https://creativecommons.org/licenses/by/4.0/ This content is distributed under the terms of the Creative Commons Attribution 4.0 International license.

10.1128/mbio.02191-22.4 FIG S1 **Fluoxetine and Amitriptyline displayed the greatest antimicrobial activity when E. coli were grown in YPD.** (A) Spot test of E. coli ATCC25922 in the presence of fluoxetine (Flu) or amitriptyline (Ami) in three standard microbial nutrient media. Overnight cultures were diluted OD600 to 0.1 and 3μl samples of serial 5-fold dilutions were spotted. Plates were incubated overnight at 37C and imaged. (B) The viability of E. coli ATCC25922 on fluoxetine and amitriptyline as measured by colony forming units (CFUs). Overnight cultures were diluted to 10-5 and 100μl spread on plates containing fluoxetine or amitriptyline or no drug control. Plates were incubated overnight at 37C and CFUs counted. Plots show the percent of CFU relative to CFUs plated on no drug plates. (C) Measurements of MICs by 96-well plate microdilution method for the indicated strains and drugs. Download FIG S1, JPG file, 0.6 MB. Copyright © 2022 Ou et al. 2022 Ou et al. https://creativecommons.org/licenses/by/4.0/ This content is distributed under the terms of the Creative Commons Attribution 4.0 International license.

Next, we challenged fluoxetine-resistant (RF) mutants with a related SSRI, sertraline, and observed that a subset of RF mutants (RF3-5) displayed resistance to sertraline but RF1 and RF2 were as sensitive as the parental strain (Fig. 1C). We challenged amitriptyline-resistant (RA) mutants with two other TCAs, nortriptyline and clomipramine, and observed that all RA mutants were resistant to nortriptyline and clomipramine compared to the parental strain (Fig. 1C). Although SSRIs and TCAs are chemically distinct we tested if the resistance mechanism was shared or unique to the class of antidepressant. Toward this end, we challenged all five RF mutants by growing them on amitriptyline containing plates and all six RA mutants by growing them on fluoxetine or sertraline containing plates. We observed that RF1, 3, 4, 5, and RA1-5 strains exhibited resistance to both drugs. These results suggest that there is a common mechanism of resistance across SSRIs and TCAs (Fig. 1C).

### Fluoxetine- and amitriptyline-resistant E. coli display resistance to antibiotics.

**FIG 2:**
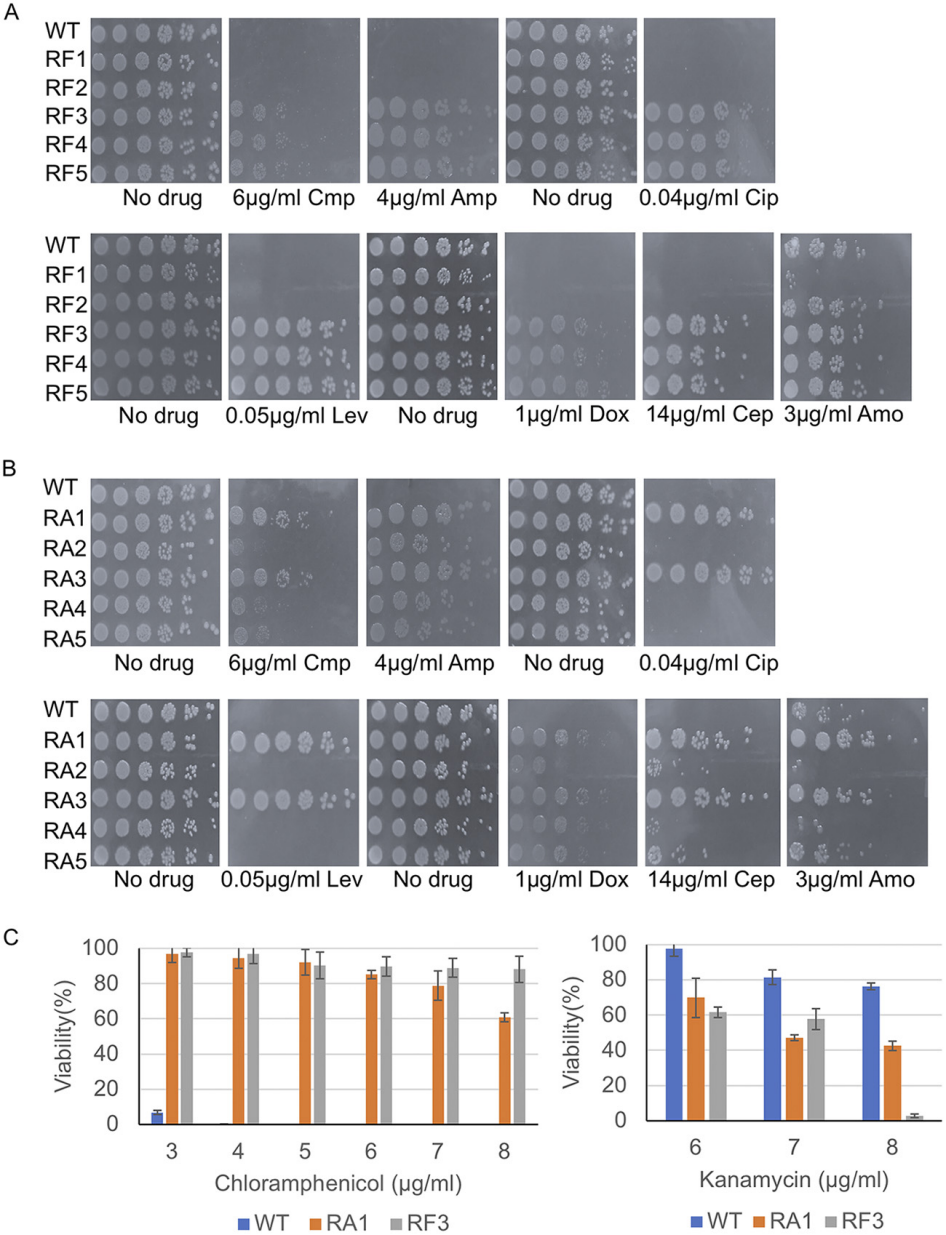
Fluoxetine- and amitriptyline-resistant mutants are resistant to multiple antibiotics. (A and B) Spot tests of RF1-5 and RA1-5 were performed to test the response to several antibiotics that target different bacterial processes (ampicillin [Amp], chloramphenicol [Cmp], ciprofloxacin [Cip], levofloxacin [Lev], amoxicillin [Amo], cephalexin [Cep], and doxycycline [Dox]) at the indicated concentrations. Spot tests were performed using single colonies of each strain, grown in overnight liquid cultures, diluted to an OD_600_ of 0.1, and 3 μL of serial 5-fold dilutions were spotted on the respective plates, incubated overnight at 37°C, and imaged. (C) The viability of the wild-type, RA1, and RF3 strains was determined on increasing doses of kanamycin and chloramphenicol. Single colonies of each strain were grown in overnight cultures, diluted, and spread on plates containing chloramphenicol (3 to 8 μg/mL), kanamycin (6 to 8 μg/mL), or no drug. Plates were incubated overnight at 37°C and colonies were counted. Plotted is the average percentage of viable cells (Viability %) calculated by dividing the number of CFU on drug plates by the CFU found on no drug control plates; error bars represent SEM for three independent cultures for each strain.

Animal and human studies report an enrichment of ARGs in rodent and patient gut microbiota when treated with SSRIs and TCAs (21, 25). Thus, we challenged our RF and RA mutants with commonly used antibiotics to test if these mutants displayed collateral resistance to antibiotics. We chose antibiotics, which included several that are within the top most prescribed in the clinic (50), that inhibited cell wall synthesis (ampicillin, cephalexin, amoxicillin), protein synthesis (chloramphenicol, kanamycin, doxycycline), transcription (rifampin), and replication (ciprofloxacin, levofloxacin). We observed that RF3-5 were more resistant to nearly all antibiotics tested compared with the RF1, RF2, and the parental strains (Fig. 2A). However, RF2-5 displayed similar sensitivities to rifampicin as the parental strain, while RF1 displayed enhanced sensitivity (Fig. 2A; Fig. S2A). Similarly, amitriptyline-resistant mutants RA1 and RA3 displayed resistance to nearly all antibiotics tested except rifampicin (Fig. 2B; Fig. S2A). RA2, 4, and 5 strains displayed similar sensitivities to nearly all antibiotics but were more resistant to ampicillin and doxycycline compared to the parental strain (Fig. 2B; Fig. S2A). We observed that the RF3-5 and RA1, RA3 mutants which displayed resistance to most of the antibiotics were hypersensitive to kanamycin (Fig. S2A). In contrast, mutant strains (RF2, RA2, RA4, RA5) maintained similar sensitivity as the parental strain to all antibiotics, including kanamycin (Fig. 2A and B).

10.1128/mbio.02191-22.5 FIG S2 Characterization of RF, RA, RC mutants on antibiotics and expression levels of acrA, acrB, tolC, marA mRNAs in RA11 and RA12 mutants. (A) Spot tests of RF and RA mutants challenged with rifampicin or kanamycin. (B) Measurments of mRNA levels for the indicated genes in RA11 and RA12 mutant strains, normalized to wild-type (WT). Shown are the average values and SEMs from three biological replicates for each strain. (C) Spontaneously isolated chloramphenicol resistant (RC) mutants display resistance to ampicillin (Amp) but hypersensitivity to hygromycin B (Hyg B). Spot tests of RC mutants subjected to different doses of ampicillin or hygromycin B. (D) The viability of WT E. coli strain (ATCC 25922) on kanamycin and chloramphenicol. Overnight cultures were diluted to 10-5 and 100μl spread on agar plates containing kanamycin (7-10 μg/ml) or chloramphenicol (2-5 μg/ml), or no drug. Plates were incubated overnight at 37C and the colony-forming unit (CFUs) are plotted relative to no drug CFUs. For all spot tests, the corresponding overnight cultures were diluted to OD600 of 0.1 and 3μl samples of serial 5-fold dilutions spotted on plates. Plates were incubated overnight at 37C and imaged. Download FIG S2, JPG file, 0.7 MB. Copyright © 2022 Ou et al. 2022 Ou et al. https://creativecommons.org/licenses/by/4.0/ This content is distributed under the terms of the Creative Commons Attribution 4.0 International license.

To determine the MICs of antidepressants and antibiotics for a subset of resistant mutants compared to wild-type, we used resazurin-based 96-well plate microdilution assays Elshikh et al. (51). We determined the MIC for each antibiotic as the first concentration in which the culture remains blue after resazurin treatment (Fig. S1C). The RA1 strain’s MIC for fluoxetine was 220 μM and for amitriptyline was 440 μM compared with the wild-type strain’s MIC of 140 μM and 320 μM for fluoxetine and amitriptyline, respectively. The RF3 strain’s MIC for fluoxetine was 200 μM and for amitriptyline was 460 μM. Both RA1 and RF3 strains displayed reduced susceptibility to most antibiotics (1.5- to 4-fold increase in MIC). The RA2 and RF2 strains, displayed nearly the same MICs to all antibiotics tested as wild-type. The largest difference was in RA2, which displayed a 4-fold increase in MIC for doxycycline and 2-fold increase for levofloxacin compared to wild-type (Fig. S1C). These results confirm the responses observed in the spot tests showing that RF3 and RA1 mutants are, to different degrees, broadly resistant across antidepressants and antibiotics while others have more limited resistance to only antidepressants such as RA2 and RF2 mutants.

### Chloramphenicol-resistant E. coli display cross-resistance to SSRIs and TCAs.

**FIG 3:**
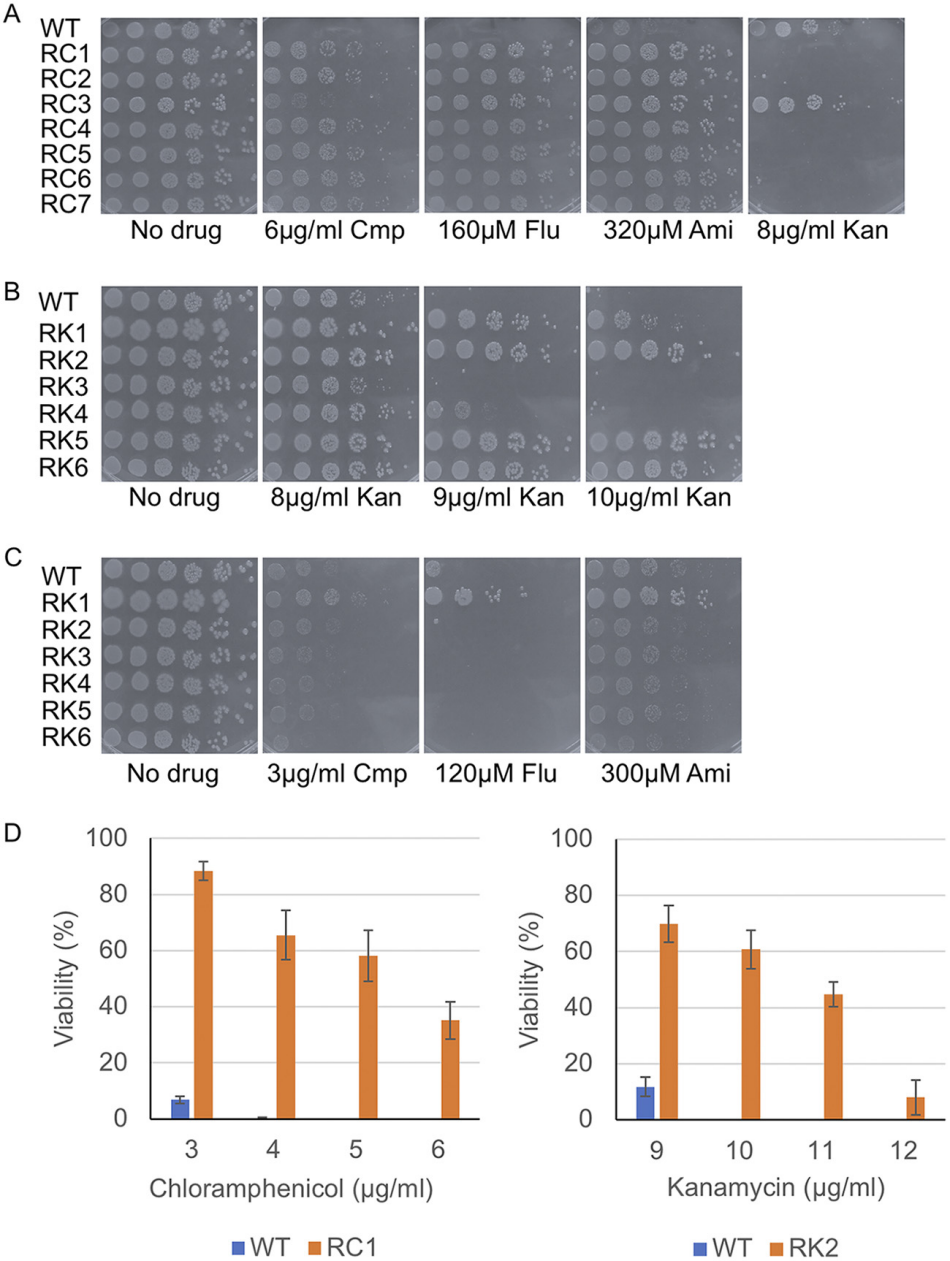
Spontaneously isolated chloramphenicol but not kanamycin-resistant mutants display resistance to fluoxetine and amitriptyline. (A) Spot tests of chloramphenicol-resistant (RC) mutants show all RC mutants have greater resistance to chloramphenicol (Cmp) (RC3 displays the weakest resistance) and to fluoxetine (Flu) and amitriptyline (Ami) compared with wild-type cells. However, RC1, 2, and 4 to 7 are sensitive to kanamycin (Kan) compared with wild-type cells. (B) Spot tests of kanamycin-resistant (RK) mutants show RK1, 2, and 4 to 6 are more resistant to kanamycin than the wild-type cells. (C) Spot tests of kanamycin-resistant (RK) mutants show all RK mutants, except RK1, have similar sensitivities to chloramphenicol, fluoxetine, and amitriptyline compared with wild-type cells. RK1 displays resistance to all three drugs. For all spot tests, single colonies of each strain were grown in overnight liquid cultures, diluted to an OD_600_ of 0.1, and 3 μL of serial 5-fold dilutions were spotted on the respective plates, incubated overnight at 37°C, and imaged. (D) The viability of wild-type, RC1, and RK2 strains on chloramphenicol and kanamycin. Single colonies of each strain were grown in overnight cultures, diluted, and spread on plates containing chloramphenicol (3 to 6 μg/mL), kanamycin (9 to 12 μg/mL), or no drug. Plates were incubated overnight at 37°C and colonies were counted. Plotted is the average percentage of viable cells (Viability %) calculated by dividing the number of CFU on drug plates by the CFU found on no drug control plates; error bars represent SEM for three independent cultures for each strain.

Previous reports show that spontaneous resistance to one antibiotic can lead to simultaneous cross-resistance and/or collateral sensitivity to another antibiotic(s) (52, 53). We observed that RF3-5 and RA1 and RA3 strains were resistant to chloramphenicol (Fig. 2C; Fig. S2A) but hypersensitive to kanamycin suggesting that fluoxetine and amitriptyline might select for similar resistance mechanisms as chloramphenicol. To test this possibility, we isolated spontaneous mutants that displayed resistance to chloramphenicol (RC) followed by testing for resistance to fluoxetine and amitriptyline. Seven RC mutants were isolated and their resistance to chloramphenicol compared with the parental strain was confirmed (Fig. 3A). The RC mutants displayed some differences in their degree of resistance to chloramphenicol (e.g., RC3). When RC mutants were tested against fluoxetine and amitriptyline, all displayed greater resistance to both antidepressants compared with the parental strain (Fig. 3A). When RC mutants were tested against kanamycin, they were all more sensitive compared with the parental strain except for RC3 (Fig. 3A and D; Fig. S2C). The RC mutants were also more resistant to ampicillin as was observed for the RF and RA mutants (Fig. S2C). These results suggest that resistance to fluoxetine, amitriptyline, and chloramphenicol have a common mechanism.

10.1128/mbio.02191-22.6 FIG S3 Replica plating tests of initial fluoxetine resistant mutants from chemical-genomic screen of the KEIO library. Mutants were first grown as patches on no drug YPD plates (top row) then replica plated on drug containing plates (bottom row) and screened for resistance. Below plate images are the names of the genes deleted in the indicated strains. Spot tests on bottom show differential resistance of KEIO mutants grown on sertraline (Ser). Download FIG S3, JPG file, 0.5 MB. Copyright © 2022 Ou et al. 2022 Ou et al. https://creativecommons.org/licenses/by/4.0/ This content is distributed under the terms of the Creative Commons Attribution 4.0 International license.

10.1128/mbio.02191-22.7 FIG S4 Replica plating tests of initial amitriptyline resistant mutants from chemical-genomic screen of the KEIO library. Mutants were first grown as patches on no drug YPD plates (top row) then replica plated onto drug containing plates (bottom row) and screened for resistance. Below the plate images are the names of the genes deleted in the indicated strains. Download FIG S4, JPG file, 0.6 MB. Copyright © 2022 Ou et al. 2022 Ou et al. https://creativecommons.org/licenses/by/4.0/ This content is distributed under the terms of the Creative Commons Attribution 4.0 International license.

Previously, it was reported that resistance to aminoglycosides such as kanamycin resulted in collateral sensitivity to chloramphenicol (53). Isolating mutants resistant to antidepressants (fluoxetine and amitriptyline) that are also hypersensitive to kanamycin prompted us to test if spontaneously resistant kanamycin mutants would display collateral sensitivity to antidepressants. We isolated spontaneous kanamycin-resistant (RK) mutants that were able to survive on 10 μg/mL of kanamycin, a dose that typically causes a complete loss in viability in the parental strain (Fig. S2D). Six mutants were isolated and tested for resistance on several doses of kanamycin. Only four of the six RK mutants showed greater resistance to kanamycin than the parental strain (Fig. 3B and D). We then tested all six mutants for sensitivity to fluoxetine and amitriptyline. We observed only a subtle increase in sensitivity to fluoxetine, amitriptyline, and chloramphenicol compared with the parental strain (Fig. 3C). These results suggest that the resistance to kanamycin does not lead to collateral sensitivity to fluoxetine or amitriptyline.

### Mutant strains that are resistant to antidepressants and antibiotics carry mutations in the transcriptional repressor marR and the lon protease.

**FIG 4:**
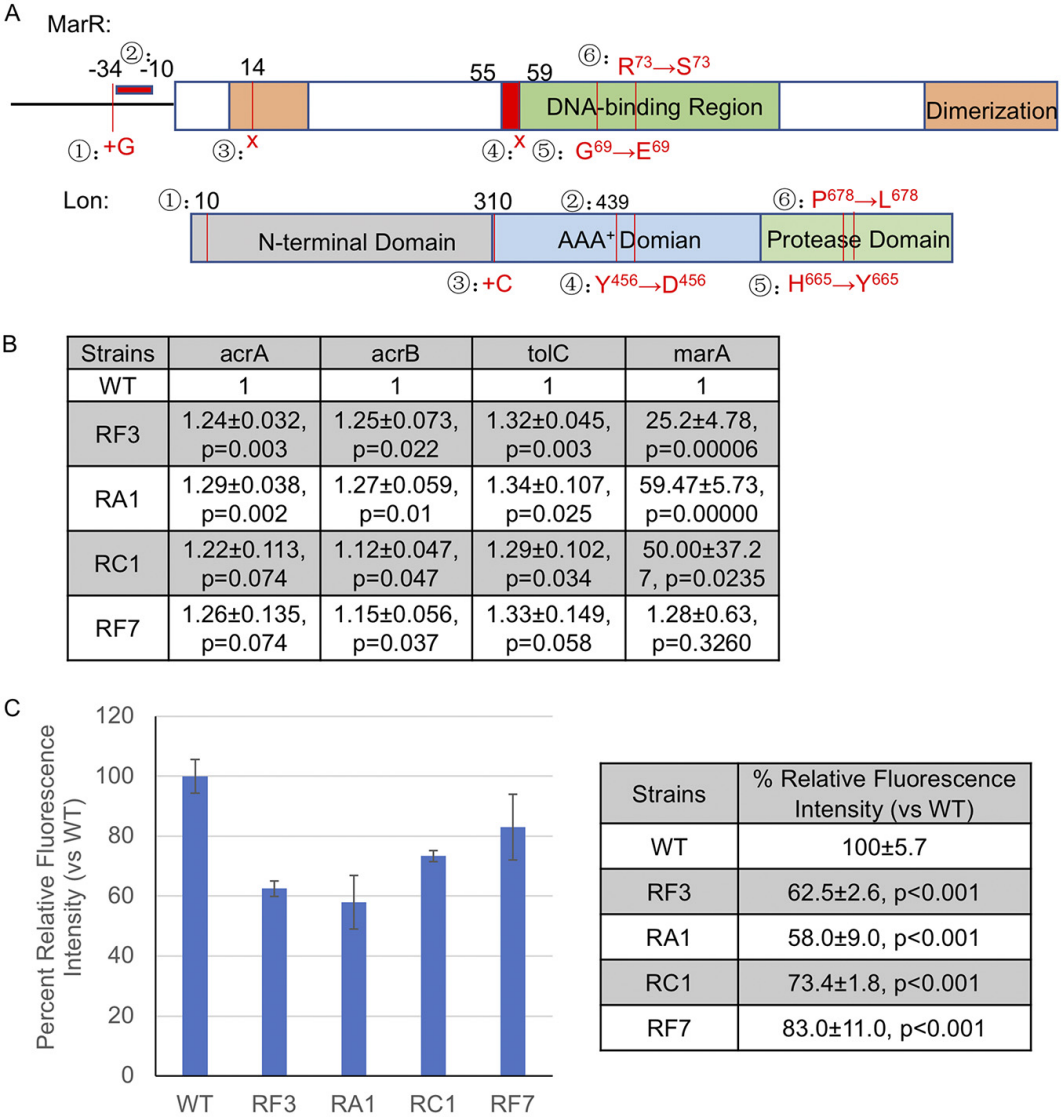
Mutants broadly resistant to antidepressants and antibiotics have increased expression in acrA, acrB, and tolC genes and display greater efflux activity. (A) Location and description of mutations in marR and lon identified in resistant (RA, RC, RF) mutants. (mutations in MarR) The boxed area represents the coding sequence (144 amino acids) and the black line to the left represents the upstream regulatory promoter region. Green shading represents the DNA-binding region of MarR (aa 55 to 100). Brown shading represents the regions involved in MarR dimerization (aa 10 to 22 and 123 to 144). Numerated are the mutations identified in resistant strains: ① Insertion of a G in the 34 nt upstream of marR; ② 20 nt deletion upstream of marR ORF starting at −10; ③ Nonsense mutation L14* (TTG→TAG); ④ Nonsense mutation E59* (GAA→TAA); ⑤ G69E (CCT→CTT); ⑥ R73S (CGT→AGT); (mutations in Lon) The boxed area represents the coding sequence (784 amino acids). Gray shading represents sequence for N-terminal (aa 1 to 309). Blue shading represents sequence coding for the AAA+ domain of lon (aa 309 to 585). Green shading represents sequence coding for the protease domain of lon (aa 585 to 784). Numerated are the mutations identified in resistant strains: ① Δ1 bp (29/2355 nt); ② Δ1 bp (1316/2355 nt); ③ +C (931/2355 nt); ④ Y456D (TAC→GAC); ⑤ H665Y (CAC→TAC); ⑥ P678L (CCG→CTG). (B) Levels of acrA, acrB, tolC, and marA mRNAs in resistant mutants versus wild-type cells. Measurements of mRNAs are all normalized to wild-type, values are averages and SEMs analyzed by Student's *t* test to determine the corresponding *P*-values. (C) Efflux activity measured as a function of Hoescht 33342 accumulation. Plotted (left) is the mean fluorescence intensity relative to wild-type (set to 100%) at 30 min and error bars represent SEMs. Values of the same measurements in the table (right) and analyzed by Student's *t* test to determine the corresponding *P* values. In (B) and (C), four independent biological replicates were performed for each strain.

To determine the genetic basis for the drug resistance in RF, RA, RC, and RK mutants, we isolated genomic DNA from 21 strains representing subsets of each group of resistant mutants and two wild-type strains, and performed whole-genome sequencing. We compared the genotypes of all 23 strains with the drug phenotypes (Table S2) using linear regression and Fisher’s exact test. As shown in Table 1, the linear regression analysis between the phenotypes and genotypes indicates that mutations in the marR coding and in upstream regulatory regions are significantly associated (*P* < 0.05) with seven out of the eight drug-resistant phenotypes, and has a *P* value of 0.055 for the kanamycin sensitivity. This association for marR was also observed by Fisher’s exact test (*P* < 0.05) in seven out of the eight drug-resistance phenotypes, and has a *P* value of 0.069 for the kanamycin sensitivity (Table 2). We identified nonsense, missense, and short deletion mutations (i.e., four codon deletion) in the marR gene in RF, RA, and RC mutants but not in RK mutants, which displayed wild-type sensitivities to antidepressants and chloramphenicol (Table 3). Nonsense mutations were observed in RA3 and RC1 which are predicted to truncate or nearly eliminate the entire MarR protein. Based on the crystal structure of MarR the point mutations and small four amino acid in-frame deletions are predicted to disrupt DNA binding (54, 55) (Fig. 4A). The missense and small deletion changes in RA1, RA9; RF3 to 5; RF7; and RC1 to 2 are located in the first helix of the helix-turn-helix DNA-binding motif (Fig. 4A), or in the DNA-recognition helix in RA3 (Fig. 4A), or have mutations in the promoter region in RA10, RA11. The lack of mutations in marR in RK mutants is consistent with an absence of cross-resistance to fluoxetine, amitriptyline, and chloramphenicol (Fig. 3C). The RF1, RF2, RA2, and RA4 mutants that were resistant primarily to the antidepressant and ampicillin did not carry mutations in marR (Table 3). Therefore, the mutants which displayed resistance across antidepressants and antibiotics also carried mutations predicted to confer a loss-of-function in MarR activity, an important regulator of the AcrAB-TolC pump and antibiotic resistance (47–49). We observed missense and insertion mutations in lon in four strains (RA8, RA12, RF6, RF8) but this was not found to be significantly associated with any phenotypes in our linear regression analysis or Fisher’s exact test. However, as we show in the chemical-genomic screens, deletions in lon were found to confer broad resistance across antidepressants and antibiotics.

**TABLE 1:** Linear regression analysis of whole-genome sequences from spontaneous resistant strains^*a*^

Gene	Flu^*b*^	Ami	Amp	Cmp	Kan	Hyg B	Rif	Cip
DR76_1637				0.043				
DR76_1801	0.022	0.022		0.046	0.023			
DR76_2414					0.012			
DR76_3669	0.080	0.080		0.076	0.002			
DR76_3697	0.022	0.022		0.046	0.023			
DR76_4	0.045	0.045						
DR76_4101					0.003			
DR76_4583	0.080	0.080		0.020				
DR76_686	0.012	0.012				0.040		
gspJ					0.024	0.040		
Lon				0.092				
marR	0.023	0.023	0.002	0.015	0.055	0.000	0.000	0.000
Pic						0.040		
secA					0.024			

a Shown are p values for tests of association with resistance to each of the indicated drugs.

b Flu, fluoxetine; Ami, amitriptyline; Amp, ampicillin; Cmp, chloramphenicol; Kan, kanamycin; Hyg B, hygromycin B; Rif, rifampicin; Cip, ciprofloxacin.

**TABLE 2:** Fisher’s exact test of whole-genome sequences from spontaneous resistant strains^*a*^

Gene	Flu^*b*^	Ami	Amp	Cmp	Kan	Hyg B	Rif	Cip
DR76_1801	0.053	0.053			0.043			
DR76_2414					0.040			
DR76_3669					0.005			
DR76_3697	0.053	0.053			0.043			
DR76_4101					0.025			
DR76_4583				0.074				
DR76_686	0.026	0.026				0.071		
marR	0.039	0.039	0.009	0.017	0.069	0.008	0.008	0.008
Pic						0.071		

a Shown are p values for tests of association with resistance to each of the indicated drugs.

b Flu, fluoxetine; Ami, amitriptyline; Amp, ampicillin; Cmp, chloramphenicol; Kan, kanamycin; Hyg B, hygromycin B; Rif, rifampicin; Cip, ciprofloxacin.

**TABLE 3:** Mutations identified in spontaneously resistant mutants to fluoxetine, amitriptyline, and chloramphenicol

Strains	Mutations in marR or Ion	Mutation
RA1	marR	Δ12 bp (166 to 177/435 nt)
RA2	–^*a*^	–
RA3	marR	L14* (TTG→TAG)
RA8	Ion	H665Y (CAC→TAC)
RA9	marR	G69E (CCT→CTT)
RA10	marR	Δ20 bp:intergenic (-10/-183)
RA11	Both	marR:intergenic (-34/-178) Ion: Y456D (TAC→GAC)
RA12	Ion	P678L (CCG→CTG)
RF1	–	–
RF3	marR	Δ12 bp (166 to 177/435 nt)
RF4	marR	Δ12 bp (166 to 177/435 nt)
RF5	marR	Δ12 bp (166 to 177/435 nt)
RF6	Ion	+C (931/2355 nt)
RF7	marR	G69E (CCT→CTT)
RF8	Ion	+C (931/2355 nt)
RC1	marR	E59* (GAA→TAA)
RC2	marR	R73S (CGT→AGT)
RCK1	marR	E59* (GAA→TAA)
RCK2	marR	E59* (GAA→TAA)
RK5	–	–
RK6	–	–
RA2,4,9,10,11,12^P^^*b*^	–	–
RF1,2,^P^	–	–
RA8^P^	marR	G69E (CCT→CTT)
RF7^P^	marR	G69E (CCT→CTT)

a (–) indicates no mutation.

b ^P^ indicates the sequencing was done from marR PCR products.

10.1128/mbio.02191-22.2 TABLE S2 Scores for the growth of resistant mutants on indicated drugs used for gene association tests. Download Table S2, DOCX file, 0.02 MB. Copyright © 2022 Ou et al. 2022 Ou et al. https://creativecommons.org/licenses/by/4.0/ This content is distributed under the terms of the Creative Commons Attribution 4.0 International license.

Expression of the genes encoding for the AcrAB-TolC efflux pump is in part regulated by MarR (47–49). Previous work shows that MarR acts as a transcriptional repressor and loss-of-function mutations in marR result in upregulation of the marA regulon (56). To determine if our mutants resulted in transcriptional changes we measured the levels of acrA, acrB, and tolC mRNAs in wild-type cells and a subset of resistant mutants that carried mutations in marR (RF3: marR delta12, RA1: marR delta 12, RC1: marR E59 stop, RF7: marR G69E) (Fig. 4B). Compared with wild-type cells, RF3 and RA1 displayed a 1.24- and 1.3-fold increase in acrA expression, respectively (both *P* < 0.003). Similar increases in acrA were observed for RC1 (1.2-fold) and RF7 (1.3-fold) relative to wild-type but not statistically significant (*P* < 0.07). We observed increases in acrB that were statistically significant in all four mutants ranging from 1.1- to 1.3-fold higher than wild-type (*P* < 0.05). Expression of tolC was higher in RF3, RA1, and RC1 (~1.3-fold, *P* < 0.05) compared with wild-type and similarly for RF7, albeit just above the significance threshold (~1.3-fold, *P* < 0.06). Taken together, these results show that there is a general trend of increased expression in acrA, acrB, and tolC in the resistant mutants.

Loss-of-function mutations in marR lead to increased marA expression and are associated with reduced fitness (56). Compensatory mutations in lon or marA have been reported, which lower marA activity, to help reduce the fitness defect (57). We measured mRNA levels of marA to determine if our mutants in marR and lon are associated with changes in marA expression. Consistent with reports that marR mutations are associated with increased marA expression, RF7, RF3, RC1, and RA1 strains carrying single marR mutations expressed marA at levels ranging from 1.3- to 60-fold higher than wild-type (Fig. 4B). To examine the effects of lon, we measured marA in strains that carry mutations only in lon (RA12) or both lon and marR (RA11). We observed a significant increase (~19-fold) in marA in RA12, which carries a mutation near the catalytic C-terminal (P678L) region of lon. On the other hand, RA11, which has mutations in the upstream intergenic region of marR and in the C-terminus of lon (Y456D) displayed only ~2.8-fold increase in marA mRNA. While there is a dramatic increase in marA expression in our strains carrying marR mutations, the RF7 and RA11 strains did not show an increase to the same extent.

Next, we measured efflux activity in the same subset of RF, RA, and RC mutants using a Hoechst 33342 accumulation assay (58). Cells in log phase were treated with Hoechst dye and total fluorescence was measured in microtiter plates after 30 min. We observed that resistant mutants displayed significantly less fluorescence intensity by 30 min compared with wild-type cells (Fig. 4C). Relative to wild-type cells, RF3 and RA1 displayed the least fluorescence intensity (62.5 ± 2.6 and 58.0 ± 9.0, *P* < 0.001, respectively). There was also a significant decrease in Hoescht accumulation in RC1, RF7, and RF9 (73.4 ± 1.8, 83.0 ± 11.0, 86.6 ± 3.0, *P* < 0.001, respectively) relative to wild-type cells. These results demonstrate that the resistant mutants have higher efflux activity than wild-type cells.

### Chemical-genomic resistance screens reveal mutations in AcrAB-TolC regulators and new genes with diverse biological functions that protect E. coli from fluoxetine, amitriptyline, and antibiotics.

Toward identifying a comprehensive set of mutants resistant to fluoxetine and amitriptyline, we performed chemical-genomic screens with the KEIO library (59). We screened 3,912 deletions and identified 78 resistant mutants to fluoxetine and 49 resistant mutants to amitriptyline (Fig. S3 and S4). Secondary replica plating tests of fluoxetine- (Fig. S3) and amitriptyline-resistant mutants (Fig. S4) narrowed down the candidates to 38 genes. The confirmed mutants comprised a set of 38 genes (Table S3), including marR and lon, which was expected based on finding mutations in marR and lon in RF, RA, and RC strains. We also found marB another known regulator of the AcrAB-TolC pump (49). We performed spot test assays with this set of 38 mutants to determine the relative resistance of each mutant. We observed that all 38 mutants displayed some level of resistance to both drugs (Fig. S5). Deletions in marR, lon, and marB displayed resistance to both fluoxetine and amitriptyline as predicted. The other mutants we identified were comprised of genes with functions in a variety of processes ranging from metabolism (e.g., sad, usg), DNA replication and repair (e.g., recF), and membrane associated transporters and channels (e.g., mscM, yncD).

10.1128/mbio.02191-22.3 TABLE S3 List of fluoxetine and amitriptyline resistant mutants, including gene descriptions, isolated from the screens of the KEIO library. Download Table S3, DOCX file, 0.01 MB. Copyright © 2022 Ou et al. 2022 Ou et al. https://creativecommons.org/licenses/by/4.0/ This content is distributed under the terms of the Creative Commons Attribution 4.0 International license.

10.1128/mbio.02191-22.8 FIG S5 Spot tests of 38 resistant mutants on antidepressants and antibiotics isolated from chemical-genomic screens. Overnight cultures of indicated strains were diluted to OD600 of 0.1, and 3μl samples of serial 5-fold dilutions were spotted and grown on the indicated drug or no drug plates, incubated overnight at 37C, and imaged. Hygromycin was used in place of kanamycin as the KEIO strains carry a kanamycin resistance marker. Flu, fluoxetine; Ami, amitriptyline; Hyg B, hygromycin B, Cmp, chloramphenicol; Amp, ampicillin. Download FIG S5, JPG file, 0.7 MB. Copyright © 2022 Ou et al. 2022 Ou et al. https://creativecommons.org/licenses/by/4.0/ This content is distributed under the terms of the Creative Commons Attribution 4.0 International license.

Next, we determined if the 38 mutants were exclusively resistant to fluoxetine and amitriptyline or more broadly across SSRIs, TCAs, and antibiotics. All mutants were tested for sensitivity to hygromycin, chloramphenicol, and ampicillin (Fig. S5). We assigned scores (0 to 6) as a qualitative measure for resistance determined from our spot tests (Table 4). A score of zero indicates absence of growth even in the most concentrated first spot and a score of six indicates evidence of growth even in the most dilute (5–6) spot. We observed that the mutants could be divided into two groups: group G1 (19 mutants) conferred broad resistance across antidepressants and antibiotics (defined as scoring 3 to 6 for antidepressants and at least a score of 3 to 6 for 2 of 3 antibiotics tested), and group G2 (17 mutants) conferred resistance to mainly antidepressants (defined as scoring 3 to 6 for antidepressants and a score of less than 3 for all antibiotics tested) (Table 4). There was a third minor group comprised of yqiJ, a putative inner membrane channel, and mutL, a DNA repair protein, both displayed only mild resistance to antidepressants (Table 4). The G1 group included deletions in marR, marB, and lon which was expected based on their role in regulating expression of AcrAB-TolC genes. The genes in group G2 have diverse functions that include metabolism such as succinate semi-aldehyde dehydrogenase (sad), ascorbate degradation (ulaG), and iron ion binding (fixX) (Table S3).

**TABLE 4:** Scores for growth by spot tests of each deletion mutant treated by indicated drug at MIC90

Gene	Flu^*a*^	Ami	Hyg B	Cmp	Amp
lon	6	6	5	6	6
yfaT	4	4	5	3	3
mdaB	4	4	4	3	2
yqiJ	3	2	4	0	1
mutL	3	3	6	4	4
argE	4	4	0	3	3
hipA	6	6	2	4	1
acrZ	5	5	3	4	0
marC	4	5	3	2	1
yiiT	4	4	0	1	1
kefB	4	3	1	2	1
rsmB	6	5	4	4	4
arsC	4	5	2	4	4
yhbX	4	4	5	4	3
mscM	5	5	4	4	5
yagA	6	6	0	4	3
sufE	3	3	0	4	3
ycaK	3	5	5	2	3
yncD	6	6	4	4	4
ybjG	2	2	6	0	0
ydfS	6	6	5	3	4
rem	5	6	6	3	5
hybD	4	3	6	4	4
ydiY	5	3	5	4	4
ulaG	4	6	1	2	0
recF	6	5	5	5	5
nohA	6	6	3	4	5
usg	6	5	5	0	3
gshA	6	0	5	0	0
avtA	6	6	0	1	1
malQ	6	6	1	2	2
fixX	6	6	5	2	2
rtcR	6	3	0	0	0
marB	6	6	3	5	6
marR	5	3	3	3	3

a Flu, fluoxetine; Ami, amitriptyline; Hyg B, hygromycin B; CMP, chloramphenicol; Amp, ampicillin.

**FIG 5:**
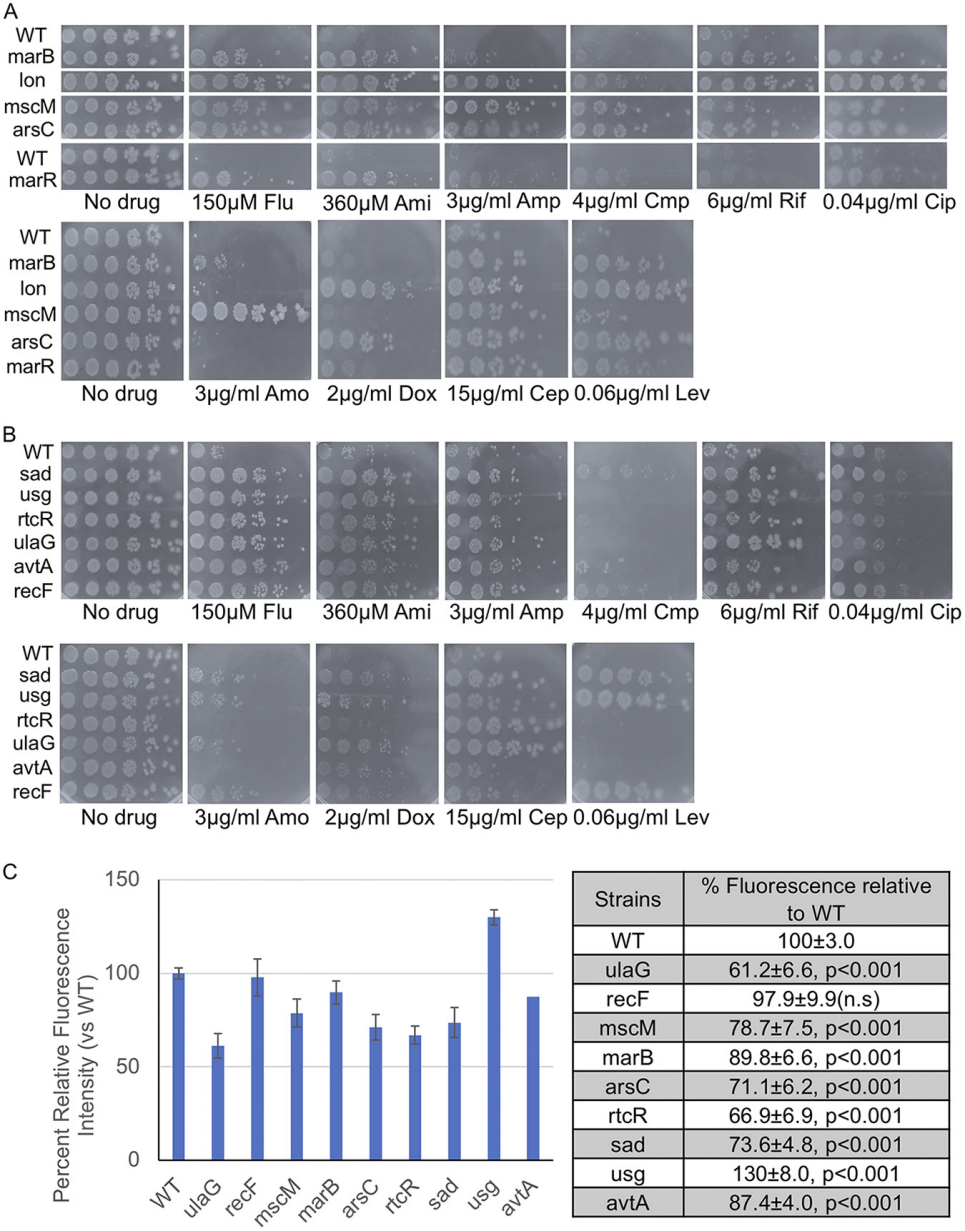
Fluoxetine- and amitriptyline-resistant mutants identified from chemical-genomic screens display differential resistance to antibiotics and some have greater efflux activity. (A and B) Spot tests of a subset of fluoxetine- and amitriptyline-resistant deletions identified from chemical-genomic screens of the Keio collection. Overnight cultures were diluted to 0.1, and 3 μL samples of serial 5-fold dilutions were spotted onto agar plates containing SSRIs (fluoxetine [Flu] and sertraline [Ser]), TCA (amitriptyline [Ami]), and antibiotics (ampicillin [Amp], chloramphenicol, [Cmp], ciprofloxacin [Cip], and rifampin [Rif], levofloxacin [Lev], amoxicillin [Amo], cephalexin [Cep], and doxycycline [Dox]) at the indicated concentrations. Plates were incubated overnight at 37°C and imaged. (C) Efflux activity was measured by Hoescht 33342 accumulation assays for the indicated deletions. Florescence intensity at 30 min is measured relative to wild-type, set at 100%. Plotted (left) is the mean fluorescence intensity relative to wild-type (set to 100%) at 30 min and error bars represent SEMs. Values of the same measurements in the table (right) and analyzed by Student's *t* test to determine the corresponding *P*-values. Four independent biological replicates were performed for each strain.

A representative set of 11 mutants from groups G1 and G2 was selected for further characterization. We performed spot test assays with the same antidepressants and antibiotics as for the initial characterization and extended the analysis to include sertraline and the same set of antibiotics tested against the RA and RF mutants (Fig. 2). We observed that mutants from G1 (marB, lon, marR, mscM, arsC, recF) displayed resistance across all drugs (Fig. 5A and Fig. S3) indicating that these mutants carried broad resistance across antidepressants and antibiotics. In contrast, mutants from G2 (sad, usg, rtcR, ulaG, avtA) displayed resistance to only the antidepressants, including sertraline but wild-type sensitivity to all antibiotics (Fig. 5B and Fig. S3). Thus, the screens identified mutants with broad resistance across antidepressants and antibiotics as well as mutants that displayed more limited resistance to the antidepressants.

### Efflux-dependent and independent mechanisms of resistance to antidepressants and antibiotics.

We tested if the mechanism of resistance observed in the 11 G1 and G2 mutants is related to increased efflux activity. First, we tested if marB and lon protease mutants, such as marR deletions (Fig. 4C), displayed higher efflux activity because they have been reported to function as negative regulators of AcrAB-TolC (47–49). We observed significant decreases in Hoescht fluorescence in marB (89.8 ± 6.6, *P* < 0.001) and lon protease (67.4 ± 3.4, *P* < 0.001) deletions compared with wild-type (100 ± 3.0) (Fig. 5C). Next we tested several of the G1 mutants (recF, mscM, arsC) displaying the greatest resistance that do not have any known role in antibiotic resistance. We observed that mutants in recF (97.9 ± 9.9) were comparable with wild-type cells (100 ± 3.0) but strains deleted in mscM (78.7 ± 7.5, *P* < 0.001) displayed significantly greater efflux activity (Fig. 5C). Several mutants in group G2 (arsC: 71.1 ± 6.2, avtA: 87.4 ± 4.0, ulaG: 61.2 ± 6.6, rtcR: 66.9 ± 6.9, all *P* < 0.001) displayed an increase in efflux activity compared with wild-type cells (Fig. 5C). In contrast, the usg mutant (130 ± 8.0) displayed greater accumulation of Hoescht compared with wild-type cells (Fig. 5C). These results suggest that resistance to antidepressants and antibiotics conferred by the gene deletions rely on efflux dependent mechanisms and in some cases there appear to be efflux-independent mechanisms.

### AcrAB-TolC is the major efflux pump that removes SSRIs and TCAs.

**FIG 6:**
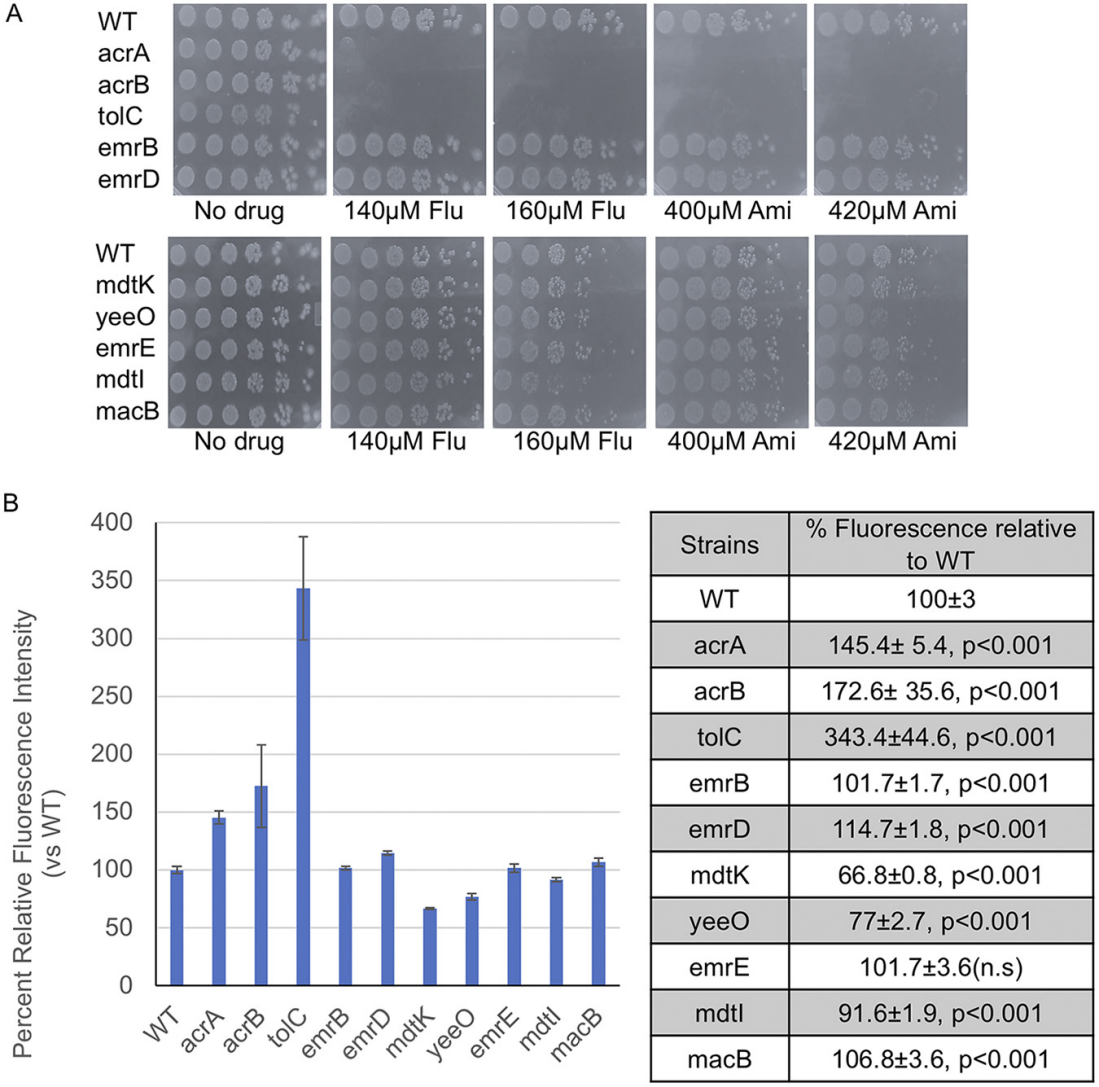
Mutations in the AcrAB-TolC pump display greater sensitivity to antidepressants. (A) Spot tests of deletions in genes encoding five classes of efflux pumps to sublethal doses of fluoxetine (Flu) and amitriptyline (Ami). Efflux pump systems: (i) RND (acrA, acrB and tolC); (ii) MFS (emrB and emrD); (iii) MATE (mdtK and yeeO); (iv) SMR (emrE and mdtI); (v) ABC (macB). Overnight cultures were diluted to an OD_600_ of 0.1 and 3 μL samples of serial 5-fold dilutions were spotted onto the indicated plates, incubated overnight at 37°C, and imaged. (B) Efflux activity was measured by Hoescht 33342 accumulation assays for the indicated deletions. Florescence intensity at 30 min is measured relative to wild-type and set at 100%. Plotted (left) is the mean fluorescence intensity relative to wild-type (set to 100%) at 30 min and error bars represent SEMs. Values of the same measurements in the table (right) and analyzed by Student's *t* test to determine the corresponding *P*-values. Four independent biological replicates were performed for each strain.

As marR, marB, and lon protease are all regulators of acrA, acrB, and tolC genes, these results suggest that export of SSRIs and TCAs may rely primarily on the AcrAB-TolC efflux pump. To test this possibility, we performed spot assays to determine the sensitivity of deletion mutants in each of the five major efflux pumps in E. coli to fluoxetine and amitriptyline: RND (acrA, acrB, tolC), MFS (emrB, emrD), MacB (macB), SMR (emrE, mdtI), and MATE (mdtK, yeeO) (47, 60). Except for mdtI, which displayed a mild sensitivity to fluoxetine, we observed that deletions in all subunits of MFS, MacB, SMR, and MATE pumps led to no observable increase in sensitivity compared with wild-type cells (Fig. 6A). On the other hand, deletions in acrA, acrB, and tolC resulted in complete loss of viability at concentrations of fluoxetine and amitriptyline which results in no discernible loss of growth in wild-type cells (Fig. 6A). Next, we tested how much each of these five efflux pumps contributes to removal of Hoescht dye in our assays. We subjected single deletions in each pump class to Hoescht accumulation assays. Mutants in the MFS, MacB, and SMR pumps resulted in negligible differences in efflux activity compared with the wild-type strain (Fig. 6B). In contrast, deletions in the MATE pump displayed greater efflux activity (Fig. 6B). Deletions in acrA, acrB, and tolC were associated with the greatest accumulation and therefore carried the least efflux activity compared with mutants in the other four pumps (Fig. 6B). Taken together, these results suggest the AcrAB-TolC pump is a major mechanism used by E. coli to remove SSRIs and TCAs and that the Hoescht accumulation assay is a good proxy for AcrAB-TolC activity.

## DISCUSSION

Overall, our work shows that fluoxetine and amitriptyline exert selection pressure for mutations that enhance activity of the AcrAB-TolC efflux pump. We found mutations in key regulators of AcrAB-TolC expression, identified new genes that contribute to efflux activities and drug resistance, and revealed overlapping resistance mechanisms between antidepressants and antibiotics. Spontaneously resistant RA and RF mutants carried mutations in marR, a transcription factor that negatively regulates expression of the AcrAB-TolC genes (49), and mutations in the lon protease, which degrades MarA, a transcriptional activator of the AcrAB-TolC genes (48, 61). RF and RA mutants isolated by fluoxetine and amitriptyline treatments displayed resistance to the SSRI sertraline, and TCAs nortiptyline and chlomipramine, suggesting that both groups of mutants were broadly resistant to SSRIs and TCAs. As AcrAB-TolC is able extrude multiple classes of antibiotics, we observed that the majority of RF and RA mutants displayed some degree of resistance to nearly all antibiotics tested. Cross-resistance and collateral sensitivity has primarily been reported between antibiotics (52, 62). Our results suggest similar relationships can occur between antidepressants and antibiotics. In particular, RF and RA mutants with the broadest resistance across antidepressants and antibiotics were always resistant to chloramphenicol but hypersensitive to kanamycin. We found that the converse also occurred in which spontaneous chloramphenicol-resistant mutants displayed resistance to fluoxetine and amitriptyline, and hypersensitivity to kanamycin. Strikingly, the chloramphenicol resistant (RC) strains also carried mutations in marR similar to the RF and RA mutants. Previous work reported mutations in spontaneous chloramphenicol-resistant mutants that were also associated with higher AcrAB-TolC activity (63). These results suggest that specific combinations of antidepressants and antibiotics may have important effects when used together or successively as they can impose selection for common mechanisms of resistance.

It is known that increased expression of efflux pumps does not necessarily confer high levels of resistance (i.e., above clinical breakpoints) (60). In several cases, our mutants (RF and RA) displayed only a 2-fold (e.g., ampicillin) to 8-fold (e.g., levofloxacin) increase in resistance. However, it is notable that highly resistant isolates in the clinic usually have mutations in the drug target and efflux related genes (60, 64). Further, marR mutations are commonly found in clinical isolates of ciprofloxacin-resistant bacteria and are thought to enhance resistance (64–69). In addition, acquisition of low-level resistance (or reduced susceptibility) may allow bacteria to survive in environments providing a chance for evolving higher level resistance (70–73). Therefore, selection for enhanced efflux activity by antidepressants may not in itself drive high levels of resistance but instead predispose bacteria to achieving clinically relevant levels of resistance.

The concentrations of SSRIs and TCAs in the gastrointestinal tract (i.e., colon) of patients is unknown. However, estimates based on factors such as drug absorption, dissolution, and transit times have been used to determine possible concentrations for SSRIs in the gut (39). Based on typical prescriptions of fluoxetine (20 to 40 mg/day) or sertraline (50 to 200 mg/day), average concentrations in the small intestine are predicted to reach 400 mg/L (1.1 mM) or 2,600 mg/L (7.6 mM) for each, respectively. These concentrations decrease in the colon to 63 mg/L (182 μM) for fluoxetine and 780 mg/L (2.3 mM) for sertraline (39). A similar estimate is not available for amitriptyline. However, if we apply the same parameters used to estimate fluoxetine concentrations to amitriptyline (taken at 10 to 150 mg/day), an average concentration of 272 mg/L (867 μM) of amitriptyline is expected to be present in the colon. These estimates do not account for any potential cumulative effects of long-term drug use or possible environmental niches within the gastrointestinal tract that could have higher or lower concentrations. While these estimates for colon concentrations (182 μM fluoxetine and 867 μM amitriptyline) need to be confirmed, they are not far off from what we used in our studies (120 to 220 μM fluoxetine and 300 to 420 μM amitriptyline). Therefore, it is possible that TCAs and SSRIs in the gut may exert selection pressure for enhanced efflux activity as we observe in *in vitro* cultures.

Our chemical-genomic screens identified deletions in marR and lon, consistent with finding loss-of-function mutations in both genes in our spontaneously isolated resistant mutants (i.e., RA, RF, RC). Furthermore, we identified a deletion in marB, another gene known to regulate AcrAB-TolC activity (60, 74–77). Additionally, the screens revealed a new group of 35 genes that contribute to antidepressant and antibiotic resistance. Notably, several deletions were in metabolism-related genes (e.g., sad, usg, argE) raising the possibility that some metabolic activities can influence efflux activity. We also identified mutations related to DNA metabolism (i.e., recF). Both MarR and MarA have been reported to regulate genes important for DNA repair and lipid trafficking suggesting the possibility that these processes may be one target of fluoxetine and amitriptyline (78). In follow-up experiments with a subset of 11 gene deletions, only three mutants (recF, usg, ulaG) did not have higher efflux activity compared with the wild-type strain. The other eight deletions all had higher efflux activity, including three known regulators of AcrAB-TolC (i.e., marR, marB, and lon). The remaining five mutants are associated with a variety of functions, including osmotic regulation (mscM), redox (arsC, sad, avtA), and outer membrane regulation (yncD). These results suggest that some physiological processes may regulate efflux activity, a possibility supported by reports showing that pumps such as AcrAB-TolC have roles in exporting bacterial metabolites and maintaining normal physiology (79–81).

Identifying AcrAB-TolC regulators in both screens underscores the importance of the AcrAB-TolC efflux pump in exporting fluoxetine and amitriptyline and other SSRIs and TCAs. Furthermore, we showed that deletions in any component of AcrAB-TolC caused a marked loss in viability when treated with sublethal doses of fluoxetine and amitriptyline. Testing deletions in five major classes of efflux pumps showed that only deletions in the AcrAB-TolC pump genes were associated with increased sensitivity to fluoxetine and amitriptyline suggesting that most export activity is supported by the AcrAB-TolC pump. This is consistent with the recent report showing that amitriptyline and chlorpromazine, an antipsychotic phenothiazine, exert a strong selection pressure for reversion of a loss-of-function acrB allele (82). Taken together, these results show that AcrAB-TolC is a major efflux mechanism that protects cells from SSRIs and TCAs. In addition to the well-known AcrAB-TolC regulators, our work reveals potentially new regulators from diverse processes suggesting that there are multiple pathways to greater efflux conferring resistance to SSRIs and TCAs. Furthermore, resistance did not always correlate with greater efflux activity as was the case for recF, usg, and ulaG. Additional work will be required to understand how efflux-independent mechanisms confer resistance. Furthermore, the diversity of mechanisms (efflux-dependent or -independent) that confer resistance to antidepressants may be much greater than what we report here, as our studies were limited to a few parental E. coli species, representing only a small part of the much larger E. coli pan genome.

Studies in rodent models of psychiatric disorders and humans have reported a general reduction in microbial diversity in the gut microbiota when treated with antidepressants (12, 16–27). Metagenomic studies reveal an enrichment of ARGs in gut microbiota when rodents and humans are treated with SSRIs and TCAs. In particular, acrA, tolC, as well as other efflux genes are found in higher abundance in samples from antidepressant treated versus untreated animals and patients (21, 25). Our identification of mutations in genes regulating AcrAB-TolC activity in antidepressant-resistant mutants suggests that the enrichment in ARGs could be a direct result of SSRI and TCA treatment. This raises the possibility that the changes in microbial composition observed in patients and animals may in part reflect a selection pressure for RND and RND-related efflux mechanisms. Future studies will be required to determine if efflux activities influence the effects and efficacy of SSRI and TCA treatments. We also showed that spontaneously resistant mutants to chloramphenicol were simultaneously resistant to antidepressants due to selection for AcrAB-TolC activity. Recently, the selective serotonin and norepinephrine reuptake inhibitor, duloxetine, and chloramphenicol were reported to impose a selection pressure for marR mutations in E. coli (83). Thus, there is cross-resistance between antidepressants and antibiotics that may be more wide-spread and suggests that treatment by one class of drug could influence the efficacy of treatment by the other class of drug due to the changes in microbial diversity and associated resistance mechanisms. In sum, this work provides a possible explanation for the reduced diversity in the gut microbiota and ARG enrichment associated with SSRI and TCA treatment, and reveals novel genes with functions in antidepressant and antibiotic resistance as well new insights to better understand the antimicrobial mechanisms of SSRIs and TCAs.

## MATERIALS AND METHODS

### Media, strains, and chemicals.

E. coli strains were purchased from the American Type Culture Collection (ATCC25922) or Dharmacon (BW25113). Typically, strains cultured in liquid yeast peptone dextrose (YPD) media (1% yeast extract, 2% peptone, and 2% glucose) or YPD plates (2% agar) at 37°C. YPD was chosen as the media for our studies as we found that the ~MIC90 of antidepressants were lower compared with Luria Broth (LB) and Tryptic Soy Broth (TSB) without changing the MICs of antibiotics (Fig. S1A). All antidepressant and antibiotic solutions were prepared from powder stocks. All antidepressants were purchased from TCI: fluoxetine hydrochloride, sertraline hydrochloride, amitriptyline hydrochloride, nortriptyline hydrochloride, clomipramine hydrochloride. The following antibiotics were purchased from the indicated companies: ampicillin sodium (GoldBio), chloramphenicol (Sigma-Aldrich), kanamycin sulfate (IBI Scientific), rifampicin (GoldBio), and ciprofloxacin HCl (GoldBio). All were dissolved in appropriate solvent (water or ethanol) to make stocks that were then diluted into media to make agar plates or liquid cultures. Other chemicals included resazurin sodium salt (Alfa Aesar) and Hoechst 33342 (Ana Spec).

### Isolation of spontaneous resistant mutants.

To isolate resistant mutants, single colonies of E. coli were grown overnight at 37°C in 5 mL of YPD. Cultures were diluted in distilled water (1/10^5^), and 100 μL was spread onto YPD agar plates containing 120, 140, 160, and 180 μM fluoxetine or 280, 300, 320, and 340 μM amitriptyline, for antibiotics 3, 4, and 5 μg/mL chloramphenicol or 8, 9, and 10 μg/mL kanamycin, respectively, to screen for antidepressant- or antibiotic-resistant bacteria after 24 h of growth at 37°C. The number of colonies that survived on drug containing and no drug YPD plates were counted to determine viability. Candidate-resistant mutants on drug plates were isolated and retested by growth assays.

Fluctuation assays were performed as described in (84) and mutation rates estimated using Shinyflan (85). Assays were performed on 50 μg/mL rifampicin, 490 μM amitriptyline, and 200 μM fluoxetine and each drug was tested twice.

### Growth assays.

Overnight cultures of resistant candidates isolated from antidepressant or antibiotic treatments were grown in 200 μL YPD media in 96-well plates at 37°C. The next day, samples were diluted to OD_600_ of 1 followed by serial 5-fold dilutions, and 3 μL of each dilution was spotted onto no drug and drug plates containing different concentrations of antidepressants or antibiotics. Plates were incubated for 24 h at 37°C and imaged.

### RNA extraction and analysis.

Bacterial cultures inoculated with single colonies were grown overnight. To obtain log phase cultures, fresh cultures were prepared from the overnight (OD_600_ ~0.1), allowed to grow until OD_600_ ~1, and harvested by centrifugation. Total RNA was extracted from bacterial pellets using Quick-RNA Fungal/Bacterial Microprep Kit (Zymo Research) and 3.5 μg was removed, and digested with DNase I (New England BioLabs). DNase treatment mix: DNase 3 μL, Buffer 2 μL, RNA 3.5 μg, and H_2_O to volume 18 μL, incubated at 37°C for 30 min, followed by adding 2 μL of 50 mM EDTA to a final concentration of 5 mM and heat inactivated at 75°C for 10 min. A 5-μL sample was used for reverse transcription with OneTaq RT-PCR Kit (New England BioLabs) to make single-stranded cDNA with oligo(dT)12–18 as a primer, followed by PCR (95°C for 30 s, 60°C for 30 s, and 72°C for 1 min, between 30 and 35 cycles) using RedTaq DNA polymerase and the respective primers (Table S4). PCR products were analyzed by agarose gel electrophoresis and the levels of mRNA expression in all samples were adjusted to GAPDH values and normalized to the wild-type strain, and quantified by Fiji software.

10.1128/mbio.02191-22.1 TABLE S4 Primer sequences. Download Table S4, DOCX file, 0.01 MB. Copyright © 2022 Ou et al. 2022 Ou et al. https://creativecommons.org/licenses/by/4.0/ This content is distributed under the terms of the Creative Commons Attribution 4.0 International license.

### Hoechst accumulation assays.

Efflux activity was inferred by measuring accumulation of Hoescht 33342 as described in Praski Alzrigat et al. (57). Strains grown overnight were used to inoculate fresh cultures the next day and grown for another 3 h at 37°C to obtain log phase cells. Cells were collected by centrifugation and resuspended in PBS, adjusted to OD_600_ of 0.5 in 5 mL PBS, and treated with Hoechst 33342 at 2.5 μM, and 200 μL was transferred to a 96-well plate (flat-bottomed, black, supplied by Greiner Bio-one). Four biological replicates for each strain were tested and at least three technical replicates were performed on each colony. The sample plate was placed in a Tecan Infinite 200 PRO plate reader, incubated at 37°C, and fluorescence was measured at 30 min. Excel (Microsoft) was used to analyze the raw fluorescence data. Mean values for each sample was calculated accounting for background controls and normalized to wild-type.

### MIC measurements.

Resazurin-based liquid assays were performed as described in (51) to determine the MIC of antidepressants and antibiotics. Single colonies were cultured overnight and recultured the next morning for 3 h at 37°C. Samples were removed and diluted to OD = 0.001 resulting in ~5 _×105_ cells/mL, added to a 96-well plate and treated with antidepressants or antibiotics. After incubation for 24 h at 37°C, resazurin (0.015%) was added to all wells (30 μL per well), and incubated for 2 h. On completion of the incubation, columns with no color change (blue resazurin color remained unchanged) were scored as the MIC value.

### Chemical-genomic screens.

A collection of 3, 985 independent, single-gene knockout mutants in the E. coli K-12 strain BW25113 was screened for resistivity to fluoxetine and amitriptyline using a 384 floating pin E-clip style manual replicator (VP 384FP6, V&P Scientific, INC.). The replicator was cleaned as previously described in Tong et al. 2006. Prior to the screen, the collection was pinned out from 96-pin format stock plates stored in 30% glycerol at −80°C using a 96 floating pin E-clip style manual replicator onto rectangular plates (source plates) containing LB supplemented with 30 μg/mL kanamycin into a 384-pin format and grown at 37°C for 1 day. For the screen, the mutants were transferred from the source plates onto rectangular plates containing YPD without addition (control) or supplemented with either 220 μM fluoxetine (TCI prod. no.: F0750) or 420 μM amitriptyline (TCI prod. no.:A0908) using a 384-pin replicator. These doses of drug represent ~ MIC90. After touching the source plate, the plates were pinned in the following order: (i) first YPD plate, (ii) YPD + 220 μM fluoxetine, (iii) YPD + 420 μM amitriptyline, and (iv) second YPD plate and grown at 37°C for 1 day. The replicator was cleaned twice after each set of plates. Mutants that showed growth on plates with 220 μM fluoxetine were recorded as fluoxetine-resistant mutants. Mutants on plates with 420 μM amitriptyline that were larger than average on the drug plate and average-sized on the control plates were recorded as amitriptyline-resistant mutants.

Candidates from the initial screen were patched onto plates containing LB plates supplemented with 30 μg/mL kanamycin and grown at 37°C for 1 day. The strains were then replica plated onto plates containing YPD supplemented with either 220 μM fluoxetine or 420 μM amitriptyline and grown at 37°C for 1 day. Strains that grew on the drug plates were confirmed as resistant (Fig. S3 and S4) and further characterized by spot tests (Fig. S5). We included the deletion in marR even though the replica plating looked weak as this was identified in the initial screen and was found in the spontaneous resistant mutants.

### Whole-genome sequencing and analysis.

Liquid cultures from single colonies were grown overnight at 37°C and harvested by centrifugation. Total genomic DNA was extracted from bacterial pellets using Quick-DNA Fungal/Bacterial Miniprep Kit (Zymo Research) and sequenced by paired-end Illumina sequencing (SNPsaurus, Eugene, OR and MicroGen Diagnostics, Lubbock, TX). Sequencing results were analyzed by Breseq (86). Output files from Breseq were secondarily analyzed using a custom written Python script to identify the following alterations compared to the parental strain: SNP, INS, DEL, MC, and SUB in the protein coding regions (CDS), a 50-bp upstream region of the starting codon, and a 50-bp downstream region of the stop codon. The corresponding genotypes for each gene in the strains are coded as 1 if the gene contains at least one mutation, and 0 if the gene does not contain a mutation. Phenotype are coded as 0 if the strain show no growth in the corresponding condition, 1 if the strain show full-growth and full resistance to the treatment, and 0.5 if the strain show moderate growth and resistance. We annotated the genotype at each gene based on 26 sequenced E. coli strains with corresponding phenotypes. The reference E. coli genome (NCBI accession no. CP009072) was used for annotations. An R script was written to perform linear regression and Fisher’s exact test between phenotypes and genotypes. We focused on genes that mutated at least once in the 23 sequenced strains. Sequence files available on Sequence Read Archive (SRA).

### Confirming gene deletions by PCR.

Liquid cultures from single colonies were grown overnight at 37°C and harvested by centrifugation. Total genomic DNA was extracted from bacterial pellets using Quick-DNA Fungal/Bacterial Miniprep Kit (Zymo Research) and 1 μL was used for PCRs. The primers designed to amplify regions −80 to −100 bp upstream or downstream of target genes (primer sequences upon request). Run PCRs for 30 cycles, beginning with 95°C for 30s, 60°C for 30s, and 72°C for 1 min. PCR products were analyzed by agarose gel electrophoresis.
