# Supplementary material for: TCA and SSRI Antidepressants Exert Selection Pressure for Efflux-Dependent Antibiotic Resistance Mechanisms in Escherichia coli
Source: mBio. 2022 Nov 14;13(6):e02191-22. doi: 10.1128/mbio.02191-22 (PMC9765716; doi:10.1128/mbio.02191-22)
Supplement: TABLE S1 [file mbio.02191-22-s0006.docx]

| *^1^E. coli* | ^2^Mutants | Antidepressions | Concentration(μM) | Survival Rate |
| --- | --- | --- | --- | --- |
| ATCC25922 | RF1, 2 (2/6) | Fluoxetine | 160 | <1% |
| ATCC25922 | RF3,4,5 (3/7) | Fluoxetine | 140 | 1.7-2.6% |
| BW25113 | RF6,7 (2/30) | Fluoxetine | 200 | <1% |
| BW25113 | RF8 (1/34) | Fluoxetine | 200 | <1% |
| ATCC25922 | RA1,2,3 (3/9) | Amitriptyline | 280 | 15% |
| ATCC25922 | RA4,5,6 (3/6) | Amitriptyline | 300 | 9.6% |
| BW25113 | RA8-12 (5/26) | Amitriptyline | 490 | <1% |

Table. S1. Spontaneously resistant mutants to fluoxetine and amitriptyline.

^1^Note: BW25113 *E. coli* strain has a higher MIC of antidepressants than the ATCC 25922 strain

^2^Values in parentheses indicate (candidates isolated/total candidates)
