# Supplementary figures and images for: TCA and SSRI Antidepressants Exert Selection Pressure for Efflux-Dependent Antibiotic Resistance Mechanisms in Escherichia coli
Source: mBio. 2022 Nov 14;13(6):e02191-22. doi: 10.1128/mbio.02191-22 (PMC9765716; doi:10.1128/mbio.02191-22)

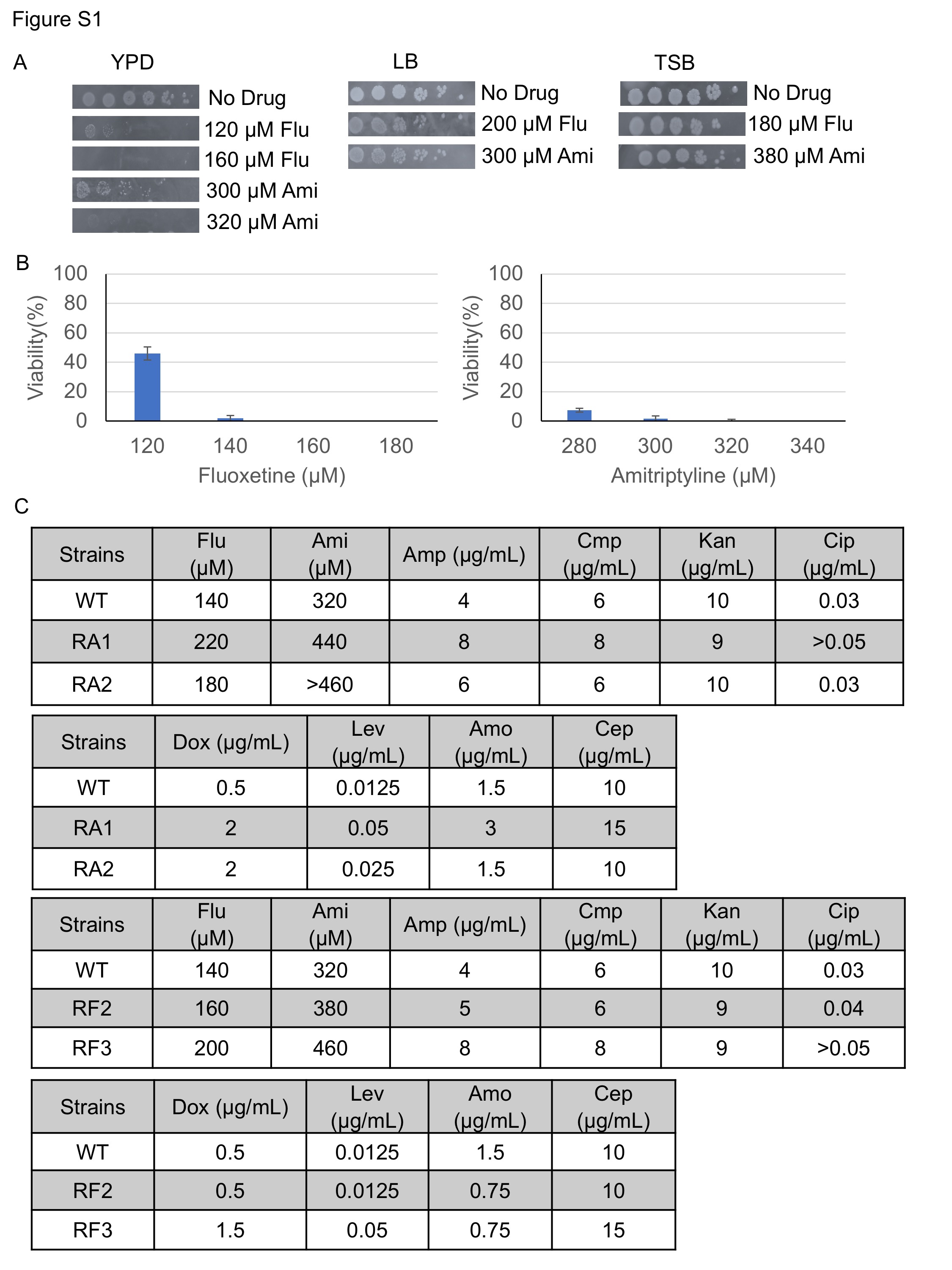

Supplement: FIG S1 [file mbio.02191-22-s0001.jpg]

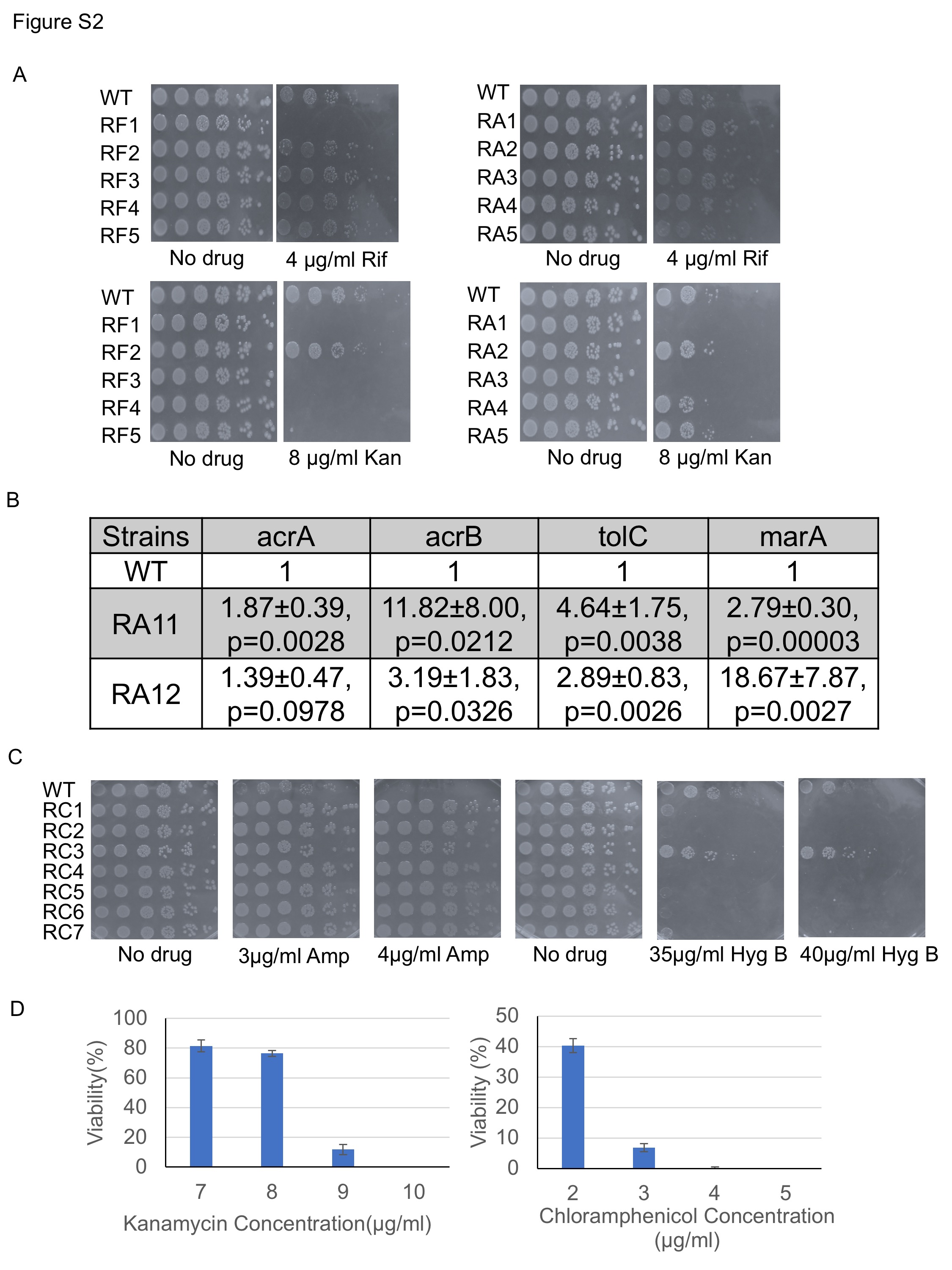

Supplement: FIG S2 [file mbio.02191-22-s0002.jpg]

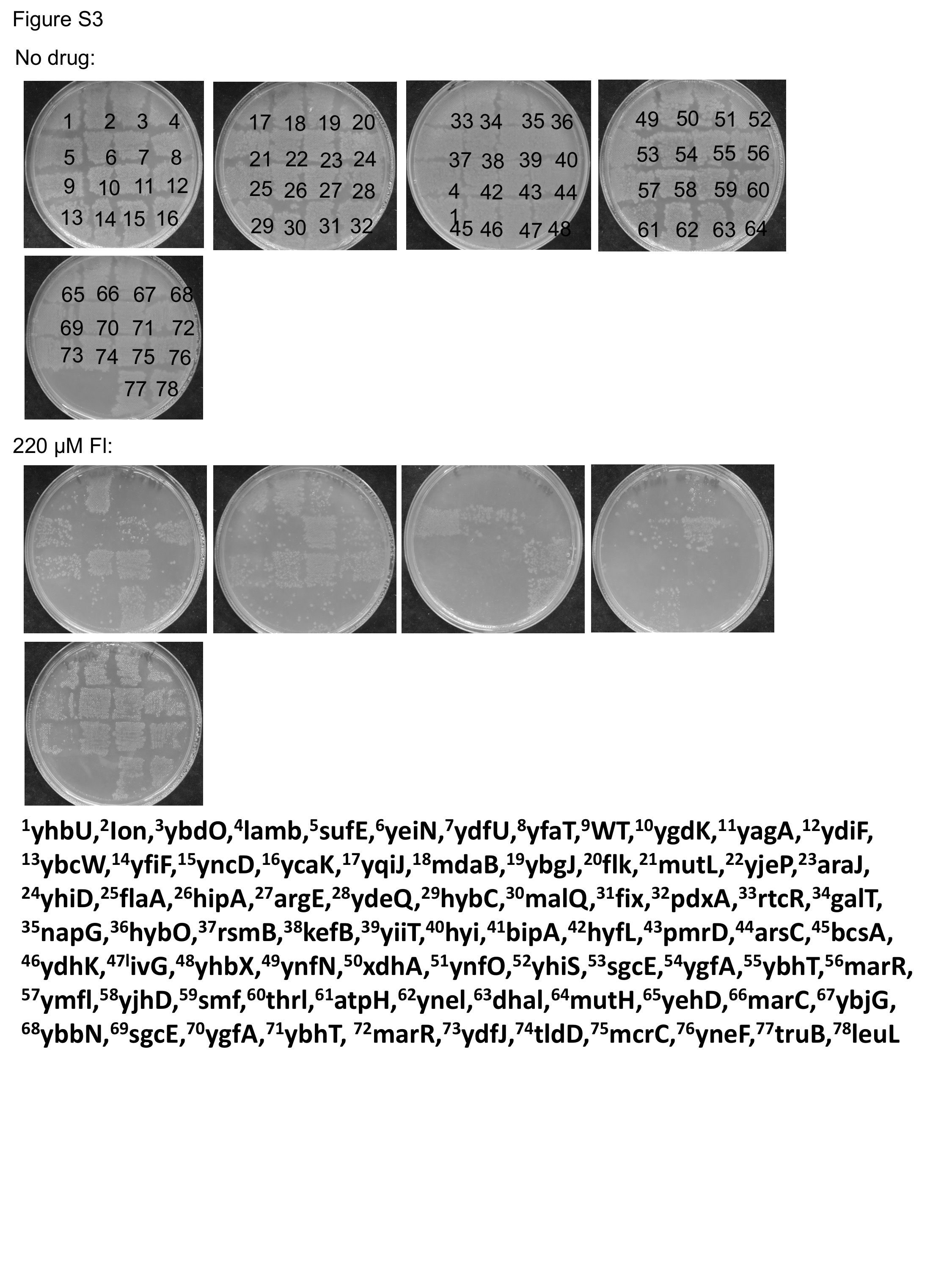

Supplement: FIG S3 [file mbio.02191-22-s0003.jpg]

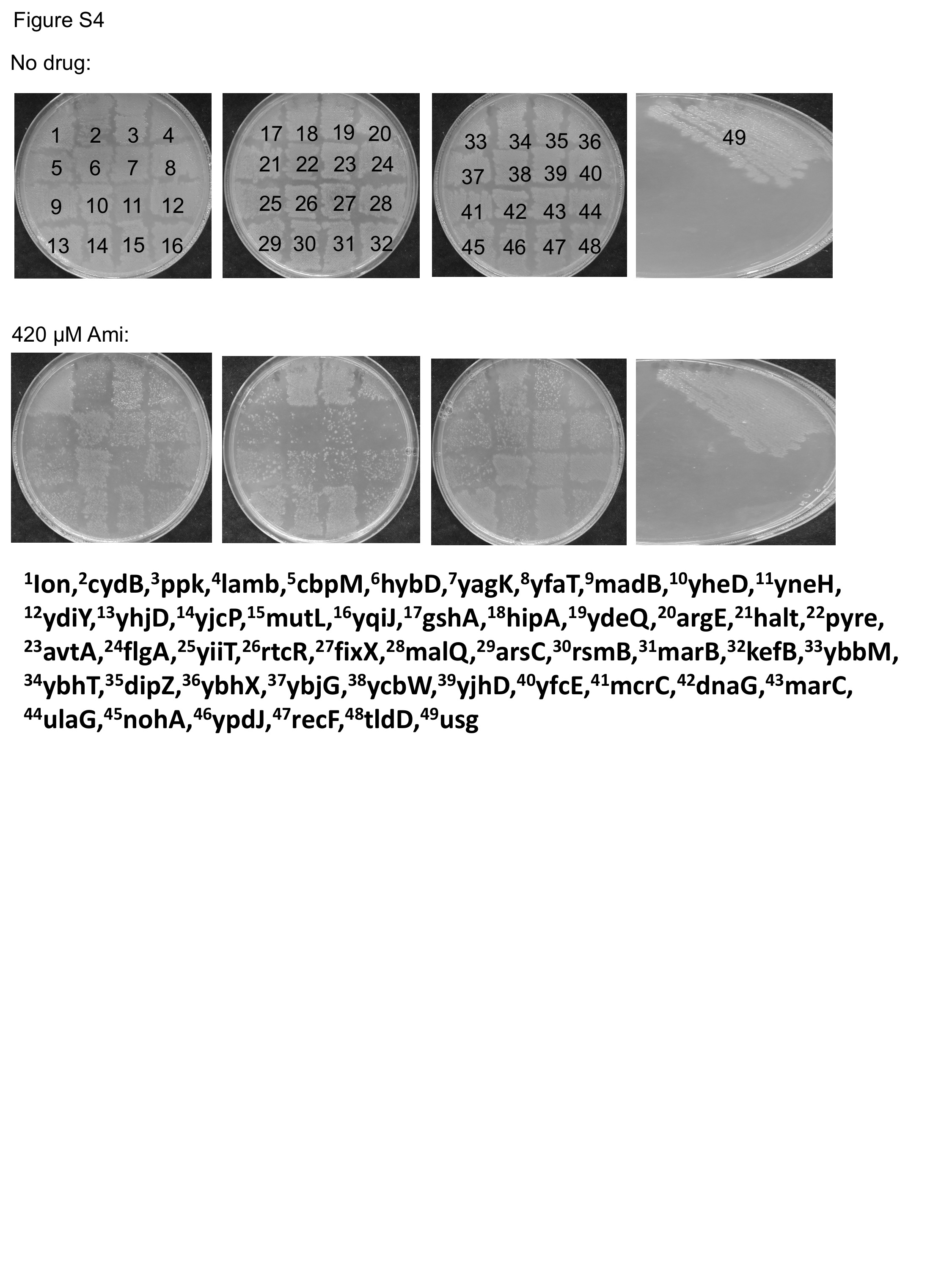

Supplement: FIG S4 [file mbio.02191-22-s0004.jpg]

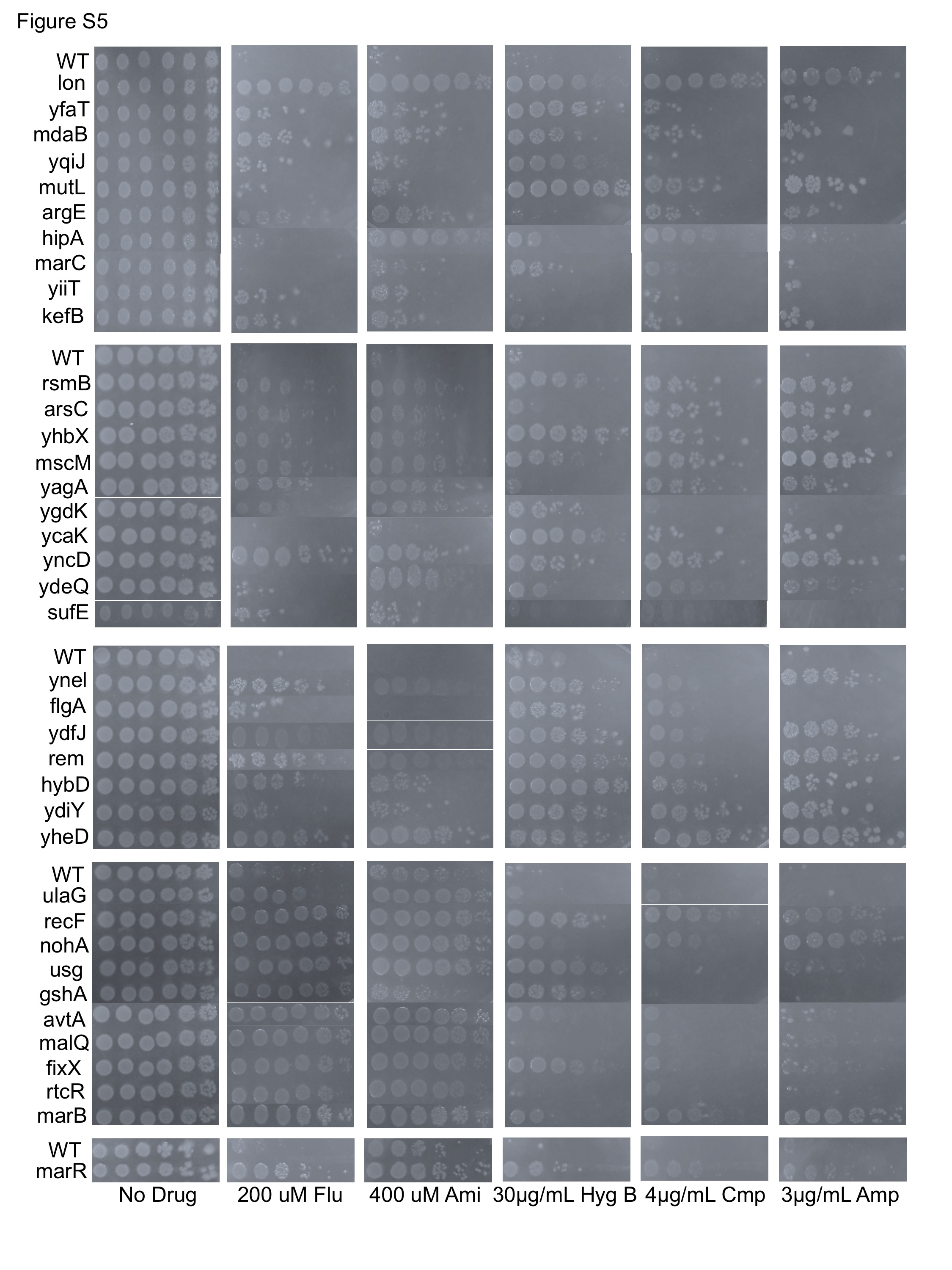

Supplement: FIG S5 [file mbio.02191-22-s0005.jpg]
