# Supplementary material for: TCA and SSRI Antidepressants Exert Selection Pressure for Efflux-Dependent Antibiotic Resistance Mechanisms in Escherichia coli
Source: mBio. 2022 Nov 14;13(6):e02191-22. doi: 10.1128/mbio.02191-22 (PMC9765716; doi:10.1128/mbio.02191-22)
Supplement: TABLE S2 [file mbio.02191-22-s0007.docx]

Table S2. Scores for growth of resistant mutants on indicated drug.

| Strains | Flu | Ami | Amp | Cmp | Kan | Hyg B | Rif | Ci |
| --- | --- | --- | --- | --- | --- | --- | --- | --- |
| WT-1 | 0 | 0 | 0 | 0 | 0 | 0 | 0 | 0 |
| WT-2 | 0 | 0 | 0 | 0 | 0 | 0 | 0 | 0 |
| RA1 | 1 | 1 | 1 | 1 | -1 | -1 | 1 | 1 |
| RA2 | 1 | 1 | 0.5 | 0.5 | 0 | 0.5 | 0 | 0 |
| RA3 | 1 | 1 | 1 | 1 | -1 | -1 | 0.5 | 1 |
| RA8 | 1 | 1 | NT | 1 | -1 | NT | NT | NT |
| RA9 | 1 | 1 | NT | 1 | -1 | NT | NT | NT |
| RA10 | 1 | 1 | NT | 1 | -1 | NT | NT | NT |
| RA11 | 1 | 1 | NT | 1 | -1 | NT | NT | NT |
| RA12 | 1 | 1 | NT | 1 | -1 | NT | NT | NT |
| RF1 | 1 | 1 | 0 | 0 | -1 | -1 | -1 | 0 |
| RF3 | 1 | 1 | 1 | 0.5 | -1 | -1 | 0 | 1 |
| RF4 | 1 | 1 | 1 | 0.5 | -1 | -1 | 0 | 1 |
| RF5 | 1 | 1 | 1 | 0.5 | -1 | -1 | 0 | 1 |
| RF6 | 1 | 1 | NT | 1 | -1 | NT | NT | NT |
| RF7 | 1 | 1 | NT | 1 | -1 | NT | NT | NT |
| RF8 | 1 | 1 | NT | 0.5 | -1 | NT | NT | NT |
| RC1 | 1 | 1 | 1 | 1 | -1 | -1 | NT | NT |
| RC2 | 1 | 1 | 1 | 1 | -1 | -1 | NT | NT |
| RCK1 | 1 | 1 | 1 | 1 | 1 | NT | NT | NT |
| RCK2 | 1 | 1 | 1 | 1 | 1 | NT | NT | NT |
| RK5 | 0 | 0 | 0 | 0 | 1 | NT | NT | NT |
| RK6 | 0 | 0 | 0 | 0 | 1 | NT | NT | NT |

1: Hypersensitive, 0: no difference compared to parental strain, 0.5-1: resistant, NT: not tested; Flu = fluoxetine, Ami = amitriptyline, Amp = ampicillin, Cmp = chloramphenicol, Kan = kanamycin, Hyg B = hygromycin B, Rif = rifampicin, Cip = ciprofloxacin
