# Supplementary material for: TCA and SSRI Antidepressants Exert Selection Pressure for Efflux-Dependent Antibiotic Resistance Mechanisms in Escherichia coli
Source: mBio. 2022 Nov 14;13(6):e02191-22. doi: 10.1128/mbio.02191-22 (PMC9765716; doi:10.1128/mbio.02191-22)
Supplement: TABLE S3 [file mbio.02191-22-s0008.docx]

Table S3. 38 Fluoxetine and amitriptyline resistant mutants and gene description

| **Gene** | **^1^Function** |
| --- | --- |
| lon | ATP-dependent protease that degrades misfolded proteins and rapidly degraded regulatory proteins |
| yfaT | may disrupt or resist host defenses |
| mdaB | NADPH: quinone oxidoreductase; modulator of drug activity B |
| yqiJ | putative inner membrane protein |
| mutL | DNA mismatch repair protein |
| argE | acetylornithine deacetylase; arginine biosynthetic pathway |
| hipA | S/T kinase mediate persistence in E. coli; inhibition of macromolecular synthesis |
| marC | inner membrane protein; involved with multiple antibiotic resistance |
| yiiT | stress protein involved in resistance to UV irradiation |
| kefB | K+:H+ antiporter |
| rsmB | 16S rRNA m5C967 methyltransferase |
| arsC | arsenate reductase |
| yhbX | putative hydrolase |
| mscM | miniconductance mechanosensitive channel |
| yagA | putative DNA-binding transcriptional regulatory |
| ygdK | part of the Suf iron-sulfur cluster assembly system |
| ycaK | NADPH degydrogenase |
| yncD | ancharacterized, putative outer membrane receptor involved with iron transport |
| ybgJ | undecaprenyl pyrophosphate phosphatase |
| ydeQ | putative fimbrial adhesin protein YdeQ |
| yneI | succinate semialdehyde dehydrogenase |
| flgA | flagellar P-ring formation protein |
| ydfJ | putative transporter YdfJ |
| rem | Qin prophage; putative S lysis protein |
| hybD | putative hydrogenase 2 maturation protease |
| ydiY | acid inducible putative outer membrane protein |
| yncD | putative TonB-dependent outer membrane receptor |
| ulaG | L-ascorbate-6-phosphate lactonase |
| *dnaG | DNA primase |
| *ybhT | multidrug efflux pump; affects substrate specificity of AcrAB-TolC |
| recF | DNA repair protein RecF |
| nohA | Qin prophage; putative prophage DNA-packaging protein NohA |
| usg | putative semialdehyde dehydrogenase Usg |
| gshA | glutamate—cysteine ligase |
| avtA | valine—pyruvate aminotransferase |
| malQ | 4-α-glucanotransferase |
| fixX | putative ferredoxin FixX |
| rtcR | DNA-binding transcriptional activator RtcR |
| marB | multiple antibiotic resistance protein MarB |
| marR | DNA-binding transcriptional repressor MarR |

^1^Gene functions were obtained from EcoCyc Database

*removed because strain genotype is dubious
