# Supplementary material for: TCA and SSRI Antidepressants Exert Selection Pressure for Efflux-Dependent Antibiotic Resistance Mechanisms in Escherichia coli
Source: mBio. 2022 Nov 14;13(6):e02191-22. doi: 10.1128/mbio.02191-22 (PMC9765716; doi:10.1128/mbio.02191-22)
Supplement: TABLE S4 [file mbio.02191-22-s0009.docx]

| Gene |  | Primer |
| --- | --- | --- |
| gap | Forward Primer | 5'-CGGTTTCGTTGTCGTACCAGG-3' |
|  | Reverse Primer | 5'-AAAGGCGTTCTGGGCTACACC-3' |
| acrA | Forward Primer | 5'-TGCGTGCACGTCTGGAAGAAG-3' |
|  | Reverse Primer | 5'-TTCAGGCTGAGCACCGCTTG-3' |
| acrB | Forward Primer | 5'-CGTTGGCGTTTATCCTCGGC-3' |
|  | Reverse Primer | 5'-CGACAGTATGGCTGTGCTCG-3' |
| tolC | Forward Primer | 5'-CGGCAGACTTACGCAATTCCG-3' |
|  | Reverse Primer | 5'-GCTCCCCATTCTTATCGGCC-3' |
| marA | Forward Primer | 5'-GCCAGGACCTGCACCAAG-3' |
|  | Reverse Primer | 5'-TGGCTGCGTGGTTTGTTCC-3' |
| marB | Forward Primer | 5'-TACTTTGATGTTCCGCCGCA-3' |
|  | Reverse Primer | 5'-TGCGGTGCTCATTATGACCG-3' |
| dnaG | Forward Primer | 5'-TAATTCCCCGAGAGCGTTGC-3' |
|  | Reverse Primer | 5'-AGCCGTTAACGCTGAAATCC-3' |
| lon | Forward Primer | 5'-GGCGTTGAATGTGGGGGA-3' |
|  | Reverse Primer | 5'-CCCCTTAATAAGGGCAAGCC-3' |
| gspA | Forward Primer | 5'-CATTCCTGCAAACCACAGGC-3' |
|  | Reverse Primer | 5'-AATGCCGCTTGCATGAATCA-3' |
| mscM | Forward Primer | 5'-GCATTTAAAACGCTGCGTCGG-3' |
|  | Reverse Primer | 5'-TTCTCTCTTGCCGGGCATCT-3' |
| arsC | Forward Primer | 5'-CAGCTGGGGATATTACTTCCG-3' |
|  | Reverse Primer | 5'-CTACGCCTGTCAGGCCTAC-3' |
| sad | Forward Primer | 5'-CGCACTTGCCGCAAGATG-3' |
|  | Reverse Primer | 5'-TCTCCAGTTGGGTCAGATCCA-3' |
| usg | Forward Primer | 5'-CTCCTGCACACTGCGGAC-3' |
|  | Reverse Primer | 5'-CAGTGCGGCATCATTGCTG-3' |
| rtcR | Forward Primer | 5'-CGTAACCGGAGATTTCCCGC-3' |
|  | Reverse Primer | 5'-TTGGCCGTAACGCGATGG-3' |
| ulaG | Forward Primer | 5'-GCTTCAGTCATGAGCTATTCCG-3' |
|  | Reverse Primer | 5'-TCAACAACAACTTCCCTGGC-3' |
| avtA | Forward Primer | 5'-CCCTAATCAGCGTTGCAGG-3' |
|  | Reverse Primer | 5'-TTTGTTTATGCCAGATGCGGC-3' |
| recF | Forward Primer | 5'-CGATATACATACCCGGGCGC-3' |
|  | Reverse Primer | 5'-TGAAATGCGAAAACGTCCGC-3' |

Table S4. Primer Sequences
